# Prophylactic phage administration provides a time window for delayed treatment of vancomycin-resistant *Enterococcus faecalis* in a murine bacteremia model

**DOI:** 10.3389/fmicb.2024.1504696

**Published:** 2025-01-24

**Authors:** Wei-Xiao Wang, Jiao-Yang Yu, Xiu-Zhen Chen, Shi-Yong Fu, Hui Li, Peng-Cheng Yi, Yun-Yao Ren, Shuang-Lin Gu, Jing-Han Gao, Jing Fan, Yan-Mei Sun, Jie Feng, Shi-Wei Wang, Wei Chen

**Affiliations:** ^1^Department of Tuberculosis, The Second Hospital of Nanjing, Affiliated to Nanjing University of Chinese Medicine, Nanjing, China; ^2^Key Laboratory of Resources Biology and Biotechnology in Western China, Ministry of Education, College of Life Sciences, Northwest University, Xi’an, China; ^3^Clinical Research Center, The Second Hospital of Nanjing, Affiliated to Nanjing University of Chinese Medicine, Nanjing, China; ^4^Department of Infectious Diseases, The Second Hospital of Nanjing, Affiliated to Nanjing University of Chinese Medicine, Nanjing, China; ^5^Department of Blood Transfusion, The Second Affiliated Hospital of Nanchang University, Nanchang, China; ^6^State Key Laboratory of Microbial Resources, Institute of Microbiology, Chinese Academy of Sciences, Beijing, China

**Keywords:** vancomycin-resistant *Enterococcus faecalis*, phage therapy, synergy, prophylactic administration, bacteremia, mouse

## Abstract

**Introduction:**

Vancomycin-resistant *Enterococcus faecalis* (VRE) poses a significant challenge in clinical settings due to its resistance to multiple antibiotics. Phage therapy offers a promising alternative to address this resistance crisis. However, critical gaps remain regarding optimal dosing, therapeutic design, and treatment timing for phage therapy targeting VRE-induced bacteremia.

**Methods:**

The biological and genomic characteristics of a novel lytic phage specific to VRE were investigated. Its *in vitro* bactericidal and antibiofilm activities were evaluated, along with its synergy with antimicrobial agents. *In vitro* safety and protective efficacy were assessed using a mouse bacteremia model. The impact of phage therapy on gut microbiota was examined through 16S rDNA gene sequencing.

**Results:**

We isolated and characterized a novel lytic phage, vB_EfaS-1017, specific to vancomycin-resistant *E. faecalis*. This phage features a circular, double-stranded DNA genome (40,766 bp), sharing 91.19% identity and 79% coverage with *Enterococcus* phage vB_EfaS_SRH2. vB_EfaS-1017 exhibited robust bactericidal and antibiofilm activity *in vitro* and demonstrated synergy with levofloxacin. Safety assessments confirmed its non-toxicity to mammalian cells and lack of hemolytic activity. In a mouse bacteremia model, phage treatment alone rescued 60% of infected mice, while combining phage with levofloxacin increased survival to 80%. Prophylactic administration of phage 24 hours prior to infection failed to prevent mortality. However, a combination of prophylactic phage administration and delayed treatment rescued 60% of mice, compared to 100% mortality in the delayed treatment alone group. Additionally, phage therapy helped maintain or restore gut microbiota balance.

**Discussion:**

These findings underscore the potential of phage-antibiotic combinations as a superior therapeutic strategy against VRE infections. The observed synergy between phages and antibiotics highlights a promising approach to overcoming bacterial resistance and improving clinical outcomes. Furthermore, prophylactic phage administration may provide a critical time window for effective delayed treatment. Further preclinical research is essential to refine phage therapy protocols for clinical application.

## 1 Introduction

*Enterococcus faecalis*, a Gram-positive, facultatively anaerobic bacterium, is a natural inhabitant of the human gastrointestinal tract. Due to its remarkable adaptability to various environmental conditions, innate resistance to commonly used antibiotics, and ability to acquire additional resistance mechanisms, *E. faecalis* thrives as a nosocomial pathogen in healthcare settings (García-Solache and Rice, 2019). Multidrug-resistant *E. faecalis* is a major contributor to a wide range of hospital-associated infections (HAIs) (Kramer et al., 2018; Brinkwirth et al., 2021; Esmail et al., 2019). Furthermore, *E. faecalis* plays a significant role in polymicrobial infections by facilitating the colonization, pathogenesis, and persistence of other pathogens, which undermines the efficacy of various antimicrobial treatments (Xu et al., 2024). Notably, *E. faecalis*-associated bacteremia is a life-threatening condition, with a mortality rate of 20–35% within 30 days (Rogers and Rice, 2023). The management of vancomycin-resistant *Enterococcus bacteremia* (VRE-B) remains a major challenge for clinicians, highlighting the urgent need to develop novel therapeutic strategies (Raza et al., 2018).

Phage therapy has experienced a resurgence due to the increasing ineffectiveness of conventional antibiotics against drug-resistant pathogens (Hatfull et al., 2022; Cobián Güemes et al., 2023; Olorundare et al., 2024). A key advantage of phages over antibiotics is their ability to lyse bacteria from within, irrespective of antibiotic resistance profiles, thereby making phage therapy a promising solution to the antibiotic resistance crisis. Phage therapy’s specificity allows it to target pathogenic bacteria without disrupting the normal microbiome. Furthermore, phages are particularly effective against developed biofilms. They can penetrate and lyse bacteria within the biofilm, facilitating the spread of progeny phages to adjacent bacterial layers (Liu et al., 2022; Cheng et al., 2017). For instance, phages have been successfully used to eradicate *E. faecalis* biofilms on urinary catheter segments (el-Atrees et al., 2022). Thus far, several successful cases of phage therapy have been reported against vancomycin-resistant *E. faecalis* in *in vivo* models (Bolocan et al., 2019; Khalifa et al., 2018). A single intraperitoneal injection of the phage EF-P29 was sufficient to protect all mice from bacteremia caused by a vancomycin-resistant *E. faecalis* strain (Cheng et al., 2017). Thus, phage therapy presents a reliable option to combat drug-resistant *E. faecalis*.

However, significant challenges still exist. First, the rapid acquisition of phage resistance by bacteria poses a barrier to phage therapy. Utilizing phage cocktails, which combine different phages, is one approach to overcoming bacterial resistance, highlighting the need for the discovery of novel phages. Second, phage-specific immunity can impair the efficacy of bacteriophage treatments targeting vancomycin-resistant *E. faecalis* (Berkson et al., 2024). A recent study has reported failure of phage therapy due to anti-phage-neutralizing antibodies, that resulted in patient mortality (Stellfox et al., 2024). This highlights a disconnect between research and clinical application, where preclinical models have not adequately guided phage implementation in humans regarding dosing, phage therapeutic design, and timing of treatment (Gómez-Ochoa et al., 2022).

In this study, a novel lytic *E. faecalis* phage was isolated and characterized both biologically and genomically. The *in vitro* bactericidal activity and the ability to inhibit biofilm formation were evaluated. The cytotoxicity and hemolytic activity of the phage were assessed. Furthermore, the combination of phage and levofloxacin was used to treat mouse bacteremia. The phage was administered at different doses and at various time points post-infection, including prophylactic use. Our preclinical study adds valuable data to the field of phage therapy against *E. faecalis* and provides helpful references for clinical application.

## 2 Methods and materials

### 2.1 Bacterial strains and growth media

All the bacterial strains used in this experiment were obtained from the clinical sample bank of the Second Hospital of Nanjing and are listed in Supplementary Table S1. *E. faecalis* and *E. faecium* were cultured in brain heart infusion (BHI) medium at 37°C, while all the other strains were cultured in Luria-Bertani (LB) medium at 37°C.

### 2.2 Isolation of bacteriophage and evaluation of host range

The phage was isolated from untreated wastewater samples collected from the Public Hygiene and Medical Center of Nanjing using vancomycin-resistant *E. faecalis* as host bacteria through the standard double-layered agar plating method. The transparent plaques were picked and transferred into 1× SM buffer (Sangon Biotech, Shanghai, China), then purified three times and stored in 20% glycerol at −80°C for long-term storage or at 4°C for periodic use. The phages were named according to the nomenclature of viruses of bacteria and archaea (Kropinski et al., 2009b).

A spot test was conducted to evaluate the host ranges of the isolated phage as described previously (Khan Mirzaei and Nilsson, 2015). Phage titer was determined using the double agar overlay plaque assay, by following established protocols (Kropinski et al., 2009a).

### 2.3 Morphology observed by transmission electron microscopy

To observe phage morphology, vB_EfaS-1017 was purified using cesium chloride (CsCl) density gradient centrifugation as described previously (Peters et al., 2022). Briefly, the phage solution was slowly added to the CsCl gradient solution, followed by centrifugation at 120,000 × *g* for 2 h at 4°C using an SW41Ti rotor in a Beckman X-100 ultracentrifuge. The phages were then collected from the corresponding density layer and transferred to a dialysis membrane (Rui Da Heng Hui, MWCO 5 kDa) for overnight dialysis. Finally, the phage morphology was observed using a Hitachi HT7700 transmission electron microscope (TEM).

### 2.4 Determination of biological features of phage

To test the thermal and pH stability of vB_EfaS-1017, the phage was suspended in the 1× SM buffer and incubated at different temperatures for 1 h, and the remaining phage titers were determined using the double-layer agar method with *E. faecalis* 10–17 as host (Kropinski et al., 2009a). To assess pH stability, the SM buffer was adjusted to different pH levels ranging from 4 to 13 using hydrochloric acid. Phage (100 μL) was mixed with adjusted buffer (900 μL) and incubated at room temperature for 1 h, after which the phage titer was measured using the double-layer agar method. To test its ultraviolet (UV) sensitivity, the phage was exposed to UV irradiation and samples were taken at various time points to determine phage viability. The experiment was performed in triplicate.

To determine the optimal multiplicity of infection (MOI) for phage vB_EfaS-1017, the phage was mixed with *E. faecalis* 10–17 at various ratios. After incubation for 4 h at 37°C with shaking, the mixture was centrifuged at 5000 rpm for 10 min. The supernatant was then filtered using a sterile 0.22 μm microporous membrane, and phage titers were measured using the double-layer agar method. The ratio that generated the highest phage titer was identified as the optimal MOI. Three independent experiments were performed.

The adsorption rate of phage vB_EfaS-1017 was determined using a log-phase culture of *E. faecalis* 10–17. Thereafter, 100 μL of 1 × 10^6^ PFU/mL phage solution was added to 1 mL of 1 × 10^8^ colony forming units (CFU)/mL bacterial culture in 10 mL of BHI broth, achieving an MOI of 0.001. The mixture was incubated at 37°C with shaking, and samples were taken at different time points. The samples were centrifuged, and the phage titer in the supernatant was determined. A phage solution without bacteria was used as a control. This experiment was also performed in triplicate.

The one-step growth curve of phage vB_EfaS-1017 was determined according to the protocol described previously (Kropinski, 2018). Overall, 100 μL of 1 × 10^6^ PFU/mL phage solution was added to 1 mL of 1 × 10^8^ CFU/mL bacterial culture in BHI broth. After a 5-min incubation to allow for adsorption, the mixture was centrifuged, and the pellet was resuspended in 50 mL of the BHI broth. The resuspended culture was incubated at 37°C with shaking, and timing commenced. Samples were taken at various intervals, and phage titers were determined using the double-layer agar method. The burst size was calculated as the ratio of the average phage titer at the stationary phase to the initial number of infected bacteria (Gadagkar and Gopinathan, 1980). The experiments were performed in triplicate.

### 2.5 Whole genome sequencing and bioinformatics analysis

The phage genome was extracted using the Solarbio DNA Viral Genome Extraction Kit to provide a high-quality template for subsequent genome sequencing and analysis. Genome sequencing was performed on the Illumina Nova 6,000 platform at Guangdong Magee Gene Biotechnology Co., Ltd. *De novo* assembly of the clean data was carried out using Megahit software (v1.1.2), and the circular genome of the phage was visualized using the CGView server database (Stothard and Wishart, 2005).

Genomic DNA annotation was conducted using Prokka software, and the function of each open reading frame (ORF) was identified manually using the Basic Local Alignment Search Tool for Proteins (BLASTp). Virulence factor analysis was performed using the Virulence Factor Database (VFDB) (Chen et al., 2005), and resistance analysis was performed using the Comprehensive Antibiotic Resistance Database (CARD) (Alcock et al., 2020). Phylogenetic tree construction was carried out using MEGA7 software (Tamura et al., 2021). Comparative BLAST analysis of phage genomes was performed using Easyfig setup (Sullivan et al., 2011). The genomic DNA sequence was deposited into the NCBI database with accession number PP894992.

### 2.6 Evaluation of bactericidal capability of phage *in vitro*

To assess the *in vitro* bactericidal efficiency of phage vB_EfaS-1017, a log-phase culture of *E. faecalis* 10–17 was prepared, washed, and suspended in 1 × PBS (pH 7.4). Phage vB_EfaS-1017 was added to the bacterial culture at a final concentration of 1 × 10^8^ CFU/mL, achieving an MOI of 0.001. The mixture was incubated at 37°C with shaking at 300 rpm. Samples were taken at various time points, and three parallel samples were set up for each time point. The remaining CFUs were counted by serial dilution and plating on BHI agar. A bacterial culture without phage served as a control. The bacterial count in the phage-treated sample was normalized to that of the control. The experiment was performed in duplicate.

To evaluate the inhibition efficiency of phage vB_EfaS-1017, a log-phase culture of *E. faecalis* 10–17 was prepared in BHI broth, diluted, and aliquoted into a 96-well plate at an initial OD_600_ of 0.01. Various doses of phage vB_EfaS-1017 were added to the bacterial cultures to achieve different MOI. Six replicates were set up for each MOI. Bacterial growth was monitored for 14 h using a plate reader, measuring optical absorbance at 600 nm. The experiment was performed in triplicate.

### 2.7 Biofilm inhibition and eradication

To determine the efficiency of biofilm inhibition by phage vB_EfaS-1017, a log-phase culture of *E. faecalis* 10–17 (OD_600_ 0.5) was prepared and aliquoted in 200 μL volumes into a 96-well clear flat-bottom plate (Corning). Various concentrations of phage vB_EfaS-1017 were added to the wells in 10 μL volumes, resulting in final phage titers ranging from 10 PFU/mL to 1 × 10^8^ PFU/mL. As a negative control, 10 μL of 1× PBS was used. To promote biofilm formation, 10% glucose was added to the wells. The plate was incubated at 37°C for 24 h, followed by crystal violet (CV) staining as described previously (Zheng et al., 2018). The optical absorbance at 570 nm was measured using a plate reader. Each sample was tested in eight wells, and these experiments were performed in triplicate. The data are presented as mean ± SD.

To evaluate the biofilm cleaning effect of phage vB_EfaS-1017, the mature biofilm of *E. faecalis* 10–17 was first developed as above, without the addition of phages. Unattached bacteria were removed, and the wells were washed twice with 1× PBS. Then, 200 μL of phages at various titers were added to each well and incubated at 37°C for 4 h. An equal volume of 1× PBS was used as a negative control. At this point, the remaining biofilms in some wells were quantified by CV staining as described above, while others were quantified by CFU counting. Bacteria within the biofilm were dislodged into 1× PBS buffer using sonication, followed by standard dilution and plating on BHI agar. These experiments were performed in triplicate, and the data are presented as mean ± SD.

To compare the biofilm clearance capabilities between phage and EDTA, 24-h biofilms of *E. faecalis* 10–17 were prepared in a Polyvinyl Chloride (PVC) 96-well plate. The mature biofilms were then, respectively, treated with 1 × 10^8^ PFU/mL of vB_EfaS-1017, 5 mM EDTA, or a combination of both at 37°C for 4 h. An equal volume of 1× PBS was used as a negative control. The remaining biofilms were quantified using CV staining as previously described. These experiments were performed in triplicate, and the data are presented as mean ± SD. To directly observe the morphology of treated biofilms, the wells were cut from the 96-well plate, immersed, and fixed in a 2.5% glutaraldehyde solution overnight. The samples were then washed three times with 1× PBS for 10 min and dehydrated in a gradient ethanol series for 15 min per step. The PVC wells were cut open and treated with an ethanol and isoamyl acetate solution (1:1) for 30 min, followed by isoamyl acetate treatment for 1 h. After critical point drying and sputter coating, the biofilms on the PVC wells were observed using scanning electron microscopy (CLARA).

### 2.8 Synergistic interaction between phage and antibiotics

To investigate the interactions between phage and antibiotics, a log-phase culture of *E. faecalis* 10–17 was prepared, diluted, and aliquoted into the 96-well plate, with approximately 1 × 10^6^ CFU per well. Gradient concentrations of antibiotics were then added to each well, with eight wells assigned to each antibiotic concentration. An equal volume of vB_EfaS-1017 in BHI broth was added to the wells, with a final concentration of 1 × 10^7^ PFU per well. Wells containing bacteria without antibiotics or phage served as the growth control. Bacterial growth was continuously monitored by measuring the optical absorbance at 600 nm using a plate reader over a 24-h period. The experiments were performed in duplicate, and representative growth curves were presented.

### 2.9 Cytotoxicity and hemolytic activity of phage

For safety evaluation *in vitro* and *in vivo*, the phage solution of vB_EfaS-1017 was first purified using the CsCl gradient centrifuge, following the same procedure described above for phage TEM observation.

The cytotoxicity of the phage suspension on normal liver cells (L02) was assessed using the Cell Counting Kit 8 (CCK8, Solarbio) following the manufacturer’s instructions. L02 cells were cultured in RPMI-1640 medium containing 20% Fetal Bovine Serum (FBS, SUNNCELL) and seeded in a 96-well plate at a density of 1 × 10^4^ cells per well. After a 24-h incubation at 37°C in a 5% CO_2_ atmosphere, the phage vB_EfaS-1017 suspension was serially diluted with 1,640 medium and added to the wells. The plate was then incubated for an additional 24 h. PBS and 2% Triton X-100 served as negative and positive controls, respectively. CCK8 solution was added to each well and incubated for 2 h before measuring the optical density at 450 nm using a plate reader. All experiments were performed in triplicate, and the data are presented as mean ± SD.

The hemolytic activity of the phage suspension was assessed according to the previously established protocol (Wang et al., 2022). Human red blood cells (RBCs) were collected from healthy volunteers, washed, and suspended in PBS. The RBC suspension was then dispensed into a 96-well V-shaped-bottom plate, and equal volumes of gradient-diluted phage vB_EfaS-1017 in PBS were added to the wells. Triton X-100 (0.1%) and PBS served as the positive control and negative control for hemolysis, respectively. After a 1-h incubation at 37°C, the supernatant was transferred to a new 96-well flat-bottom plate, and the value of OD_414_ was measured using a plate reader. Three independent experiments were performed, and the data are presented as mean ± SD.

### 2.10 Evaluation phage safety *in vivo*

In this study, specific-pathogen-free (SPF) 7-week-old female Kunming (KM) mice were utilized in all animal experiments. These mice were obtained from the Animal Multiplying Farm of Qing Long Shan in Jiangning, Nanjing. They were acclimatized for 1 week with unrestricted access to food and water and maintained under a 12-h light/dark cycle at room temperature.

Following the acclimatization period, the mice were randomly assigned to different groups, each consisting of six mice. A 200 μL suspension of 1 × 10^9^ PFU/mL phage vB_EfaS-1017 in 1× PBS was prepared daily and injected into the mice via two routes: intraperitoneal (IP) and intravenous (IV). A 200 μL injection of 1× PBS served as a negative control. The injections were administered for 1 week. Throughout the experiment, the clinical condition and body weight of the mice were monitored daily by two investigators, starting from the first injection. The scoring system was adapted from the murine sepsis score criteria (MSS) (Shrum et al., 2014), with a modification that a dead mouse was assigned a score of 5.

At the endpoint, 144 h post-injection, orbital blood samples were collected to evaluate cytokine levels using an ELISA kit. Additionally, blood samples were analyzed for liver cytotoxicity using the ARCHITECT c16000 System. Liver function test packages were manufactured by Fosun Diagnostics, including 5′-nucleotidase, adenosine deaminase, total bile acids, alkaline phosphatase, cholinesterase, lactate dehydrogenase, albumin, total protein, direct bilirubin, total bilirubin, albumin-to-globulin ratio, globulin, and indirect bilirubin.

In this study, the animals were maintained and used in accordance with the Regulations on the Administration of Experimental Animals issued by the Ministry of Science and Technology of China. All the animal experiments were approved by the Institutional Animal Care and Use Committee at Nanjing University of Chinese Medicine.

### 2.11 Determination of phage distribution and titer *in vivo*

To determine the distribution and titer of phage vB_EfaS-1017 in different organs of mice, a 200 μL suspension of 1 × 10^9^ PFU/mL phage vB_EfaS-1017 was administered to four KM mice via IP route. A 200 μL injection of 1× PBS served as a negative control. Orbital blood samples were collected from one mouse at 1 h, 6 h, 12 h, and 24 h post-injection, respectively, after which the mouse was euthanized, and its liver and spleen were harvested. Phage titers in the liver, spleen, and blood were measured using the double-layer agar method. Additionally, the total white blood cell count and neutrophil count in the blood were determined using a complete blood count examination with the Sysmex XN-2800 hematology analyzer.

### 2.12 Mouse bacteremia model

To establish a mice bacteremia model, the mice were randomly divided into seven groups, with six mice per group. Each group received a 200 μL intraperitoneal injection of varying doses of *E. faecalis* 10–17: 1 × 10^8^ CFU, 1 × 10^9^ CFU, 1 × 10^10^ CFU, 2 × 10^10^ CFU, 4 × 10^10^ CFU, and 8 × 10^10^ CFU. A seventh group received a 200 μL injection of sterile 1× PBS, serving as a negative control. The clinical condition and body weights of the mice were monitored daily by two investigators, starting from the first injection. The observation endpoint was 6 days post-infection.

### 2.13 Phage therapy against bacteremia

The mice were randomly assigned to seven groups, with ten mice per group. To establish a bacteremia model, 200 μL of *E. faecalis* 10–17 (1 × 10^10^ CFU/mL) was injected intraperitoneally. Mice injected with an equal volume of 1× PBS served as the negative control, while another group of infected, untreated mice was included as a control. At 1 h post-infection (POI), treatments were administered intraperitoneally: 2 × 10^8^ PFU of phage vB_EfaS-1017, 30 mg/kg of levofloxacin, or a combination of both. In addition, to determine the optimal timing for combination therapy, treatments were also administered immediately after infection (0 h) and at 6 h POI.

A checkpoint was established at 6 h POI to assess bacterial killing efficacy. Three mice from each group were randomly selected, euthanized, and dissected for the collection of blood, liver, and spleen samples to measure bacterial load. Survival rates were monitored every 2 h during the first 12 h and then daily. Kaplan–Meier survival curves were generated and analyzed using GraphPad Prism 9.

To investigate the cause of death of mice treated with the combination therapy, a dead mouse was dissected, and its liver was collected. Bacteria from the liver were harvested and cultured on BHI agar. Thirty colonies were picked and identified using MALDI-TOF mass spectrometry with a Clin-TOF-II instrument (BioYong, China), following the manufacturer’s recommended procedures. Component I and II reagents were procured directly from the manufacturer. The susceptibility of these 30 colonies to phage vB_EfaS-1017 was determined using a spot test, as previously described (Khan Mirzaei and Nilsson, 2015). The Levofloxacin susceptibility test was performed by the microdilution method using a 96-well plate (Benkova et al., 2020).

### 2.14 Prophylactic administration of phage therapy

The mice were randomly assigned to six groups, with seven mice per group. To assess the effect of prophylactic administration, phage vB_EfaS-1017 suspensions were administered intraperitoneally at two time points: 24 h before infection (−24 h) and 12 h before infection (−12 h), at a dose of 2 × 10^8^ per mouse. Subsequently, the bacteremia model was established as described previously. The −24 h group also received a combination treatment of 2 × 10^8^ PFU vB_EfaS-1017 and 30 mg/kg levofloxacin at 6 h post-infection. Simultaneously, a single combination treatment at 6 h post-infection served as the control for delayed treatment. Survival curves were analyzed using the Kaplan–Meier analysis in GraphPad Prism 9.

### 2.15 Flow cytometer analysis of prophylactic administration of phage

To investigate the protective mechanism of prophylactic phage administration, 30 mice were randomly assigned to two groups, with 15 mice per group. Phage vB_EfaS-1017 suspensions were administered intraperitoneally at a dose of 2 × 10^8^ PFU per mouse, while an equal volume of PBS was used as a control. Orbital blood samples were collected from three mice in each group at 0 h, 1 h, 6 h, 12 h, and 24 h post-injection. Peripheral blood mononuclear cells (PBMCs) were isolated using Ficoll density gradient centrifugation. The PBMCs were then labeled with FITC-anti-CD3 and APC-anti-HLA-DR antibodies (BD Biosciences) for 30 min at 4°C. Following labeling, the cells were washed with PBS (pH 7.4) and analyzed using a FACS Canto II flow cytometer. Data analysis was performed using FlowJo software (Treestar).

Additionally, total white blood cell counts and neutrophil counts in the blood were determined using a complete blood count examination (CBC) with the Sysmex XN-2800 hematology analyzer.

### 2.16 Determination of gut microbiota by 16 s rDNA sequencing

To evaluate whether phage therapy influences the intestinal microbiota of mice, a combination of phage and LEV was administered to the infected mice at 1 h POI. Mice in the uninfected group, infected but untreated group, and phage-treated group were euthanized, and fecal samples were harvested from the colons at 6 h POI. Each group consisted of six mice. The gut microbiota was analyzed by 16S rDNA sequencing using the Hiseq platform at Guangdong Magigene Biotechnology Co. Ltd., China.

## 3 Results

### 3.1 Isolation and identification of bacteriophage vB_EfaS-1017

**Figure 1:**
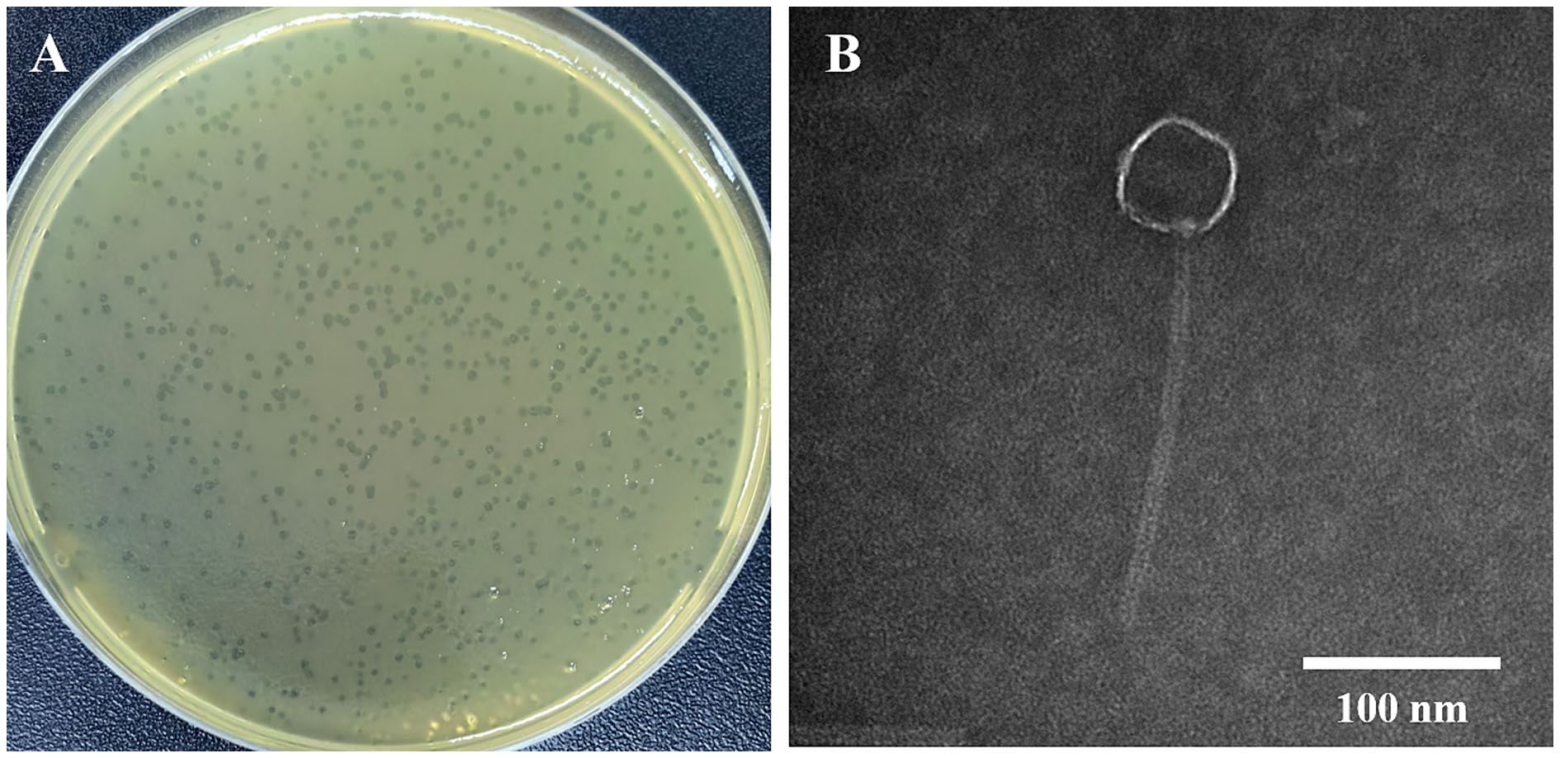
Phage plaque of vB_EfaS-1017 **(A)** and its TEM image **(B)**.

In our study, 11 vancomycin-resistant *Enterococci* (VRE) strains were used as hosts for phage isolation. Only one lytic phage was isolated from untreated medical wastewater at the Public Hygiene and Medical Center of Nanjing. This phage produced small, transparent plaques (Figure 1A). Transmission electron microscopy (TEM) revealed a long tail structure (Figure 1B), classifying it within the Siphoviridae family. This phage was then designated as vB_EfaS-1017. A spot test demonstrated that vB_EfaS-1017 had a very narrow host range, lysing only 3 out of the 11 tested *E. faecalis* strains, and none of the *E. faecium* strains or other bacteria tested (Supplementary Table S1).

**Figure 2:**
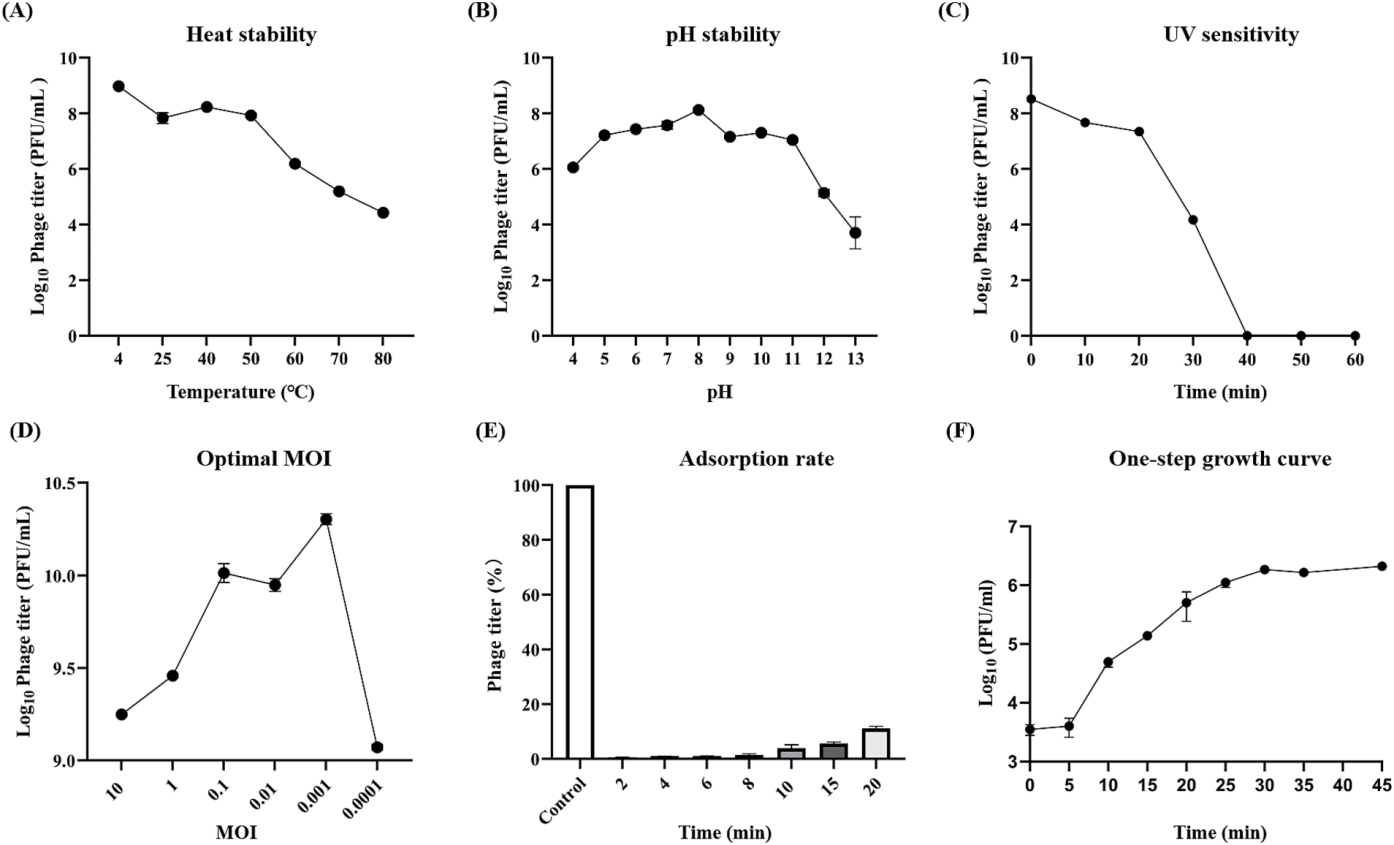
Biological features of phage vB_EfaS-1017. **(A)** Thermal stability; **(B)** pH stability; **(C)** UV sensitivity; **(D)** Optimal multiplicity of infection; **(E)** Adsorption rate; **(F)** One-step growth curve.

Heat stability assays indicated that vB_EfaS-1017 could tolerate temperatures up to 50°C, beyond which its activity decreased rapidly (Figure 2A). The pH stability assay showed that vB_EfaS-1017 remained relatively stable from pH 5.0 to pH 11.0, with optimal stability at pH 8.0 (Figure 2B). Upon UV irradiation, the phage count decreased dramatically after 20 min, and no phages were detectable by the double-layer plate method after 40 min (Figure 2C). Consequently, UV irradiation can effectively mitigate the risk of phage contamination. vB_EfaS-1017 exhibited an optimal multiplicity of infection (MOI) of 0.001, producing approximately 2 × 10^10^ PFU/mL progeny phages (Figure 2D). Adsorption assays showed complete phage attachment to host bacteria within 2 min (Figure 2E). The phage displayed a short latency period of 5 min, reaching the stationary phase at 30 min post-incubation, with a burst size of 174 PFU per cell (Figure 2F).

### 3.2 vB_EfaS-1017 is a novel *E. faecalis* phage

**Figure 3:**
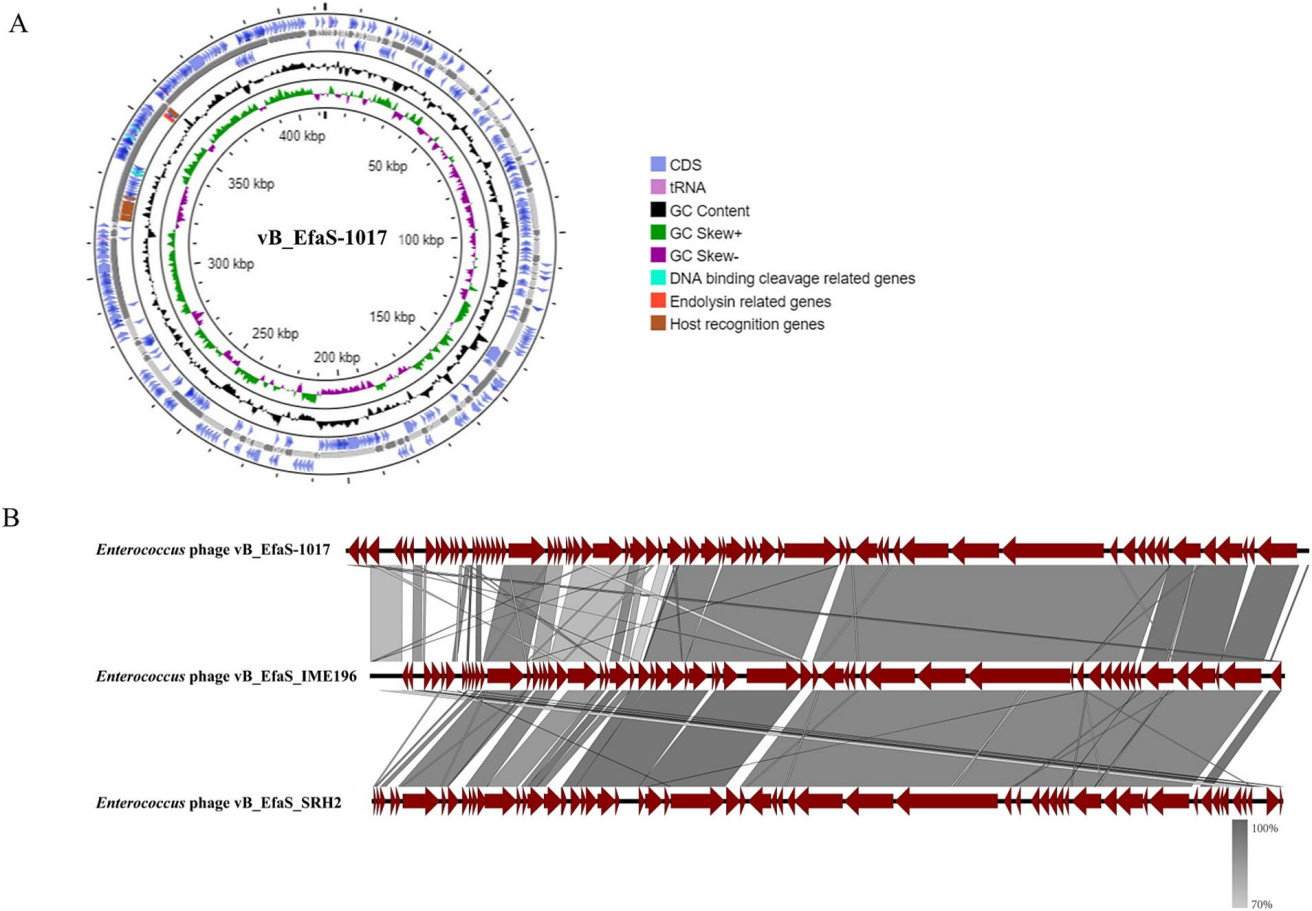
Genomic features of phage vB_EfaS-1017. **(A)** Circular map of phage vB_EfaS-1017 genome; **(B)** Comparative genome analysis of phage vB_EfaS-1017. Linear alignment of genomic sequences between the phage vB_EfaS-1017 with *Enterococcus* phage IME196 and SRH2.

Whole genome sequencing revealed that the phage vB_EfaS-1017 possessed a circular double-stranded DNA genome with a length of 40,766 bp, a GC content of 34.82%, and 65 predicted open reading frames (ORFs) (Figure 3A). A BLASTp search indicated that the phage vB_EfaS-1017 shared the highest similarity with the phage vB_EfaS_SRH2 (GenBank accession No. LC623721.1), exhibiting 91.19% identity and 79% coverage. The next most similar phage was vB_EfaS_IME196 (GenBank accession No. KT932701.1), with 88.79% identity and 79% coverage. Linear alignment of these three phage genomes demonstrated similar sequences but distinct genome organizations, suggesting the occurrence of recombination events (Figure 3B). Phylogenetic analysis of the terminase large subunit revealed that vB_EfaS-1017 was closely related to *Enterococcus* phages EnP (Supplementary Figure S1A). Furthermore, the phylogenetic tree based on the phage major capsid protein demonstrated a close relationship between vB_EfaS-1017 and *Enterococcus* phage vB_EfaS-IME196 (Supplementary Figure S1B). Collectively, these data support the classification of vB_EfaS-1017 as a novel *Enterococcus* phage.

Virulence factor analysis using the virulence factor database (VFDB) did not identify any virulence genes in the genome of phage vB_EfaS-1017. Similarly, analysis using the Comprehensive Antibiotic Resistance Database (CARD) did not detect any antibiotic resistance genes. These findings suggest that vB_EfaS-1017 has the potential to be safely administered for the treatment of *E. faecalis* infections.

### 3.3 vB_EfaS-1017 is a potent antibiofilm agent

**Figure 4:**
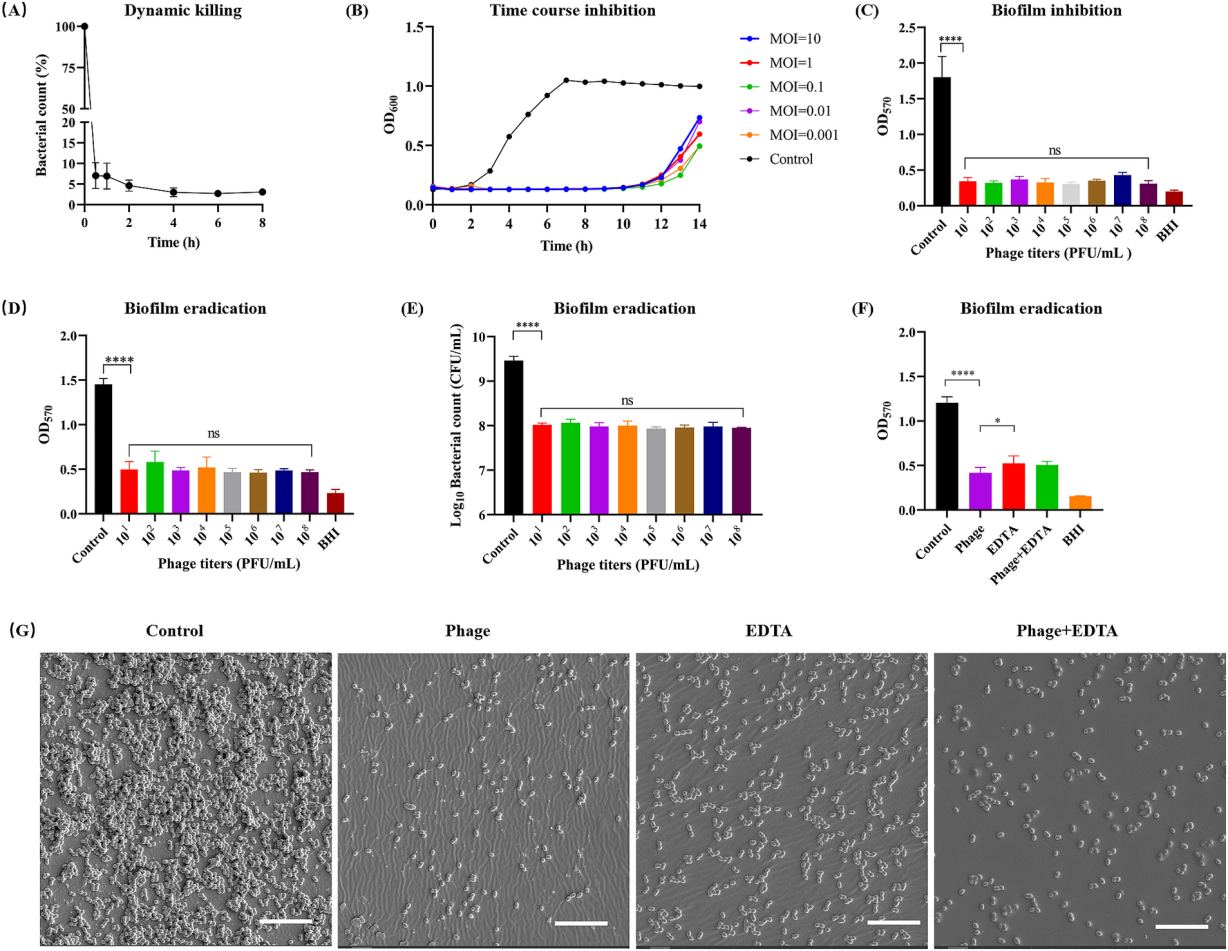
Dynamic killing and biofilm cleaning of phage vB_EfaS-1017. **(A)** *In vitro* bactericidal activity of vB_EfaS-1017 against *E. faecalis* 10–17 in 1 × PBS. **(B)** Growth inhibition of *E. faecalis* 10–17 by vB_EfaS-1017 at different MOIs in BHI broth. Bacteria without phage were used as a growth control. **(C)** Biofilm inhibition by vB_EfaS-1017. Bacteria without phage served as a control. **(D–F)** Biofilm eradication by vB_EfaS-1017. Biofilm was quantified by crystal violet (CV) staining **(D,F)** and bacterial CFU counting **(E)**. Comparison of biofilm eradication capability among phage vB_EfaS-1017, EDTA, or a combination of both **(F)**. PBS without phage was used as a negative control for biofilm clearance. **(G)** SEM images of biofilms under different treatments. Scale bar: 10 μm. Statistical analysis was performed using the one-way ANOVA with multiple comparisons. **** indicate*s p* < 0.0001; * indicates *p* < 0.05; ns indicates no statistically significant difference.

Initially, we examined the dynamic killing ability of phage vB_EfaS-1017 *in vitro*. At 37°C, this phage rapidly killed approximately 95% of *E. faecalis* cells within the first hour. Thereafter, the killing efficiency significantly decreased, with approximately 3% of bacteria remaining viable after 6 h of incubation, and no further change was observed up to 8 h (Figure 4A). A time-course inhibition assay showed that bacterial growth was completely inhibited within the first 10 h, regardless of the initial MOI. After this point, bacterial growth rebounded, suggesting the emergence of phage-resistant bacteria (Figure 4B).

Biofilm formation by *E. faecalis* significantly enhances its resistance to antimicrobial agents, making biofilm-associated infections particularly difficult to treat and posing a serious health threat despite antibiotic intervention (Sharma et al., 2023). Consequently, we investigated the capability of phage vB_EfaS-1017 to inhibit and disrupt biofilms. In the presence of phage vB_EfaS-1017, biofilm formation of *E. faecalis* 10–17 was significantly inhibited. This inhibition was independent of phage titer (Figure 4C). Similarly, vB_EfaS-1017 exhibited a potent ability to disrupt mature biofilms in a dose-independent manner (Figure 4D). Colony-forming unit (CFU) counting demonstrated that phage treatment eradicated over 90% of bacteria embedded within the biofilm (Figure 4E).

Ethylenediaminetetraacetic acid (EDTA) is known for its antimicrobial and antibiofilm properties (Finnegan and Percival, 2015). To compare the cleaning efficiency of phage vB_EfaS-1017 using EDTA, we treated mature biofilms with vB_EfaS-1017, EDTA, or a combination of both. The results indicated that 1 × 10^8^ PFU/mL of vB_EfaS-1017 showed a superior ability to clean biofilms compared to 5 mM EDTA. The combination of both agents did not exhibit an enhanced cleaning efficiency (Figure 4F). To directly assess the biofilm-cleaning efficacy, biofilms developed on a 96-well PVC plate were observed using scanning electron microscopy (SEM). Compared to the untreated biofilm control, phage treatment removed most of the bacterial cells attached to the PVC surface, better than the EDTA-treated group (Figure 4G). Therefore, phage vB_EfaS-1017 was a promising antimicrobial and antibiofilm agent against *E. faecalis*.

### 3.4 Phage vB_EfaS_10–17 exhibits synergistic interaction with levofloxacin

**Figure 5:**
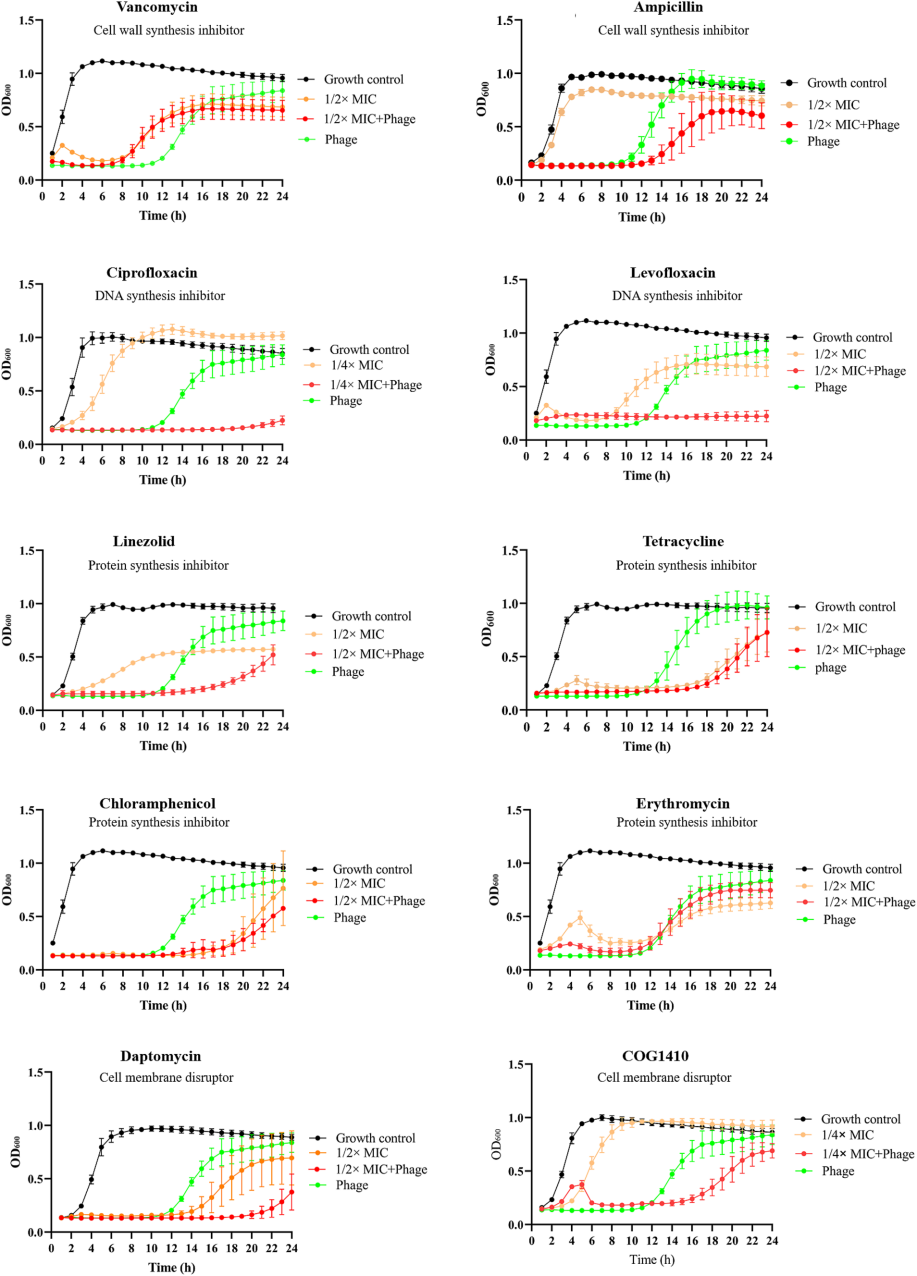
Interaction between phage vB_EfaS-1017 and various antibiotics. Antibiotics with different mechanisms of action were used. Bacteria without phage and antibiotics were set up as the growth control. OD_600_ was monitored continuously by a plate reader.

To assess whether phage vB_EfaS-1017 exhibits synergy with antibiotics against *E. faecalis*, we selected a panel of antibiotics frequently employed in clinical settings, measured their minimal inhibitory concentrations (MIC) using a microdilution assay (Supplementary Table S2) and then evaluated their interactions with the phage. Vancomycin, a glycopeptide antibiotic effective against most gram-positive microorganisms, exerts its bactericidal effect by inhibiting the polymerization of peptidoglycans in the bacterial cell wall (Koyama et al., 2012). Unexpectedly, phage vB_EfaS-1017 demonstrated antagonism with vancomycin. The combination of phage and vancomycin was less effective in inhibiting bacterial growth than phage alone. Meanwhile, no significant interactions were observed between phage vB_EfaS-1017 and tetracycline, chloramphenicol, or erythromycin (Figure 5).

Remarkably, phage vB_EfaS-1017 exhibited synergistic effects when combined with ciprofloxacin, levofloxacin, linezolid, daptomycin, and COG1410, all of which have distinct mechanisms of action. The combinations of phage and these antibiotics inhibited bacterial growth between 16 and 20 h. However, bacterial growth eventually resumed in all cases except for the combination of phage and 1/2 MIC levofloxacin, which completely inhibited bacterial growth within 24 h (Figure 5). Therefore, this combination was selected for subsequent *in vivo* phage therapy experiments.

### 3.5 Phage vB_EfaS_10–17 is safe *in vitro* and *in vivo*

**Figure 6:**
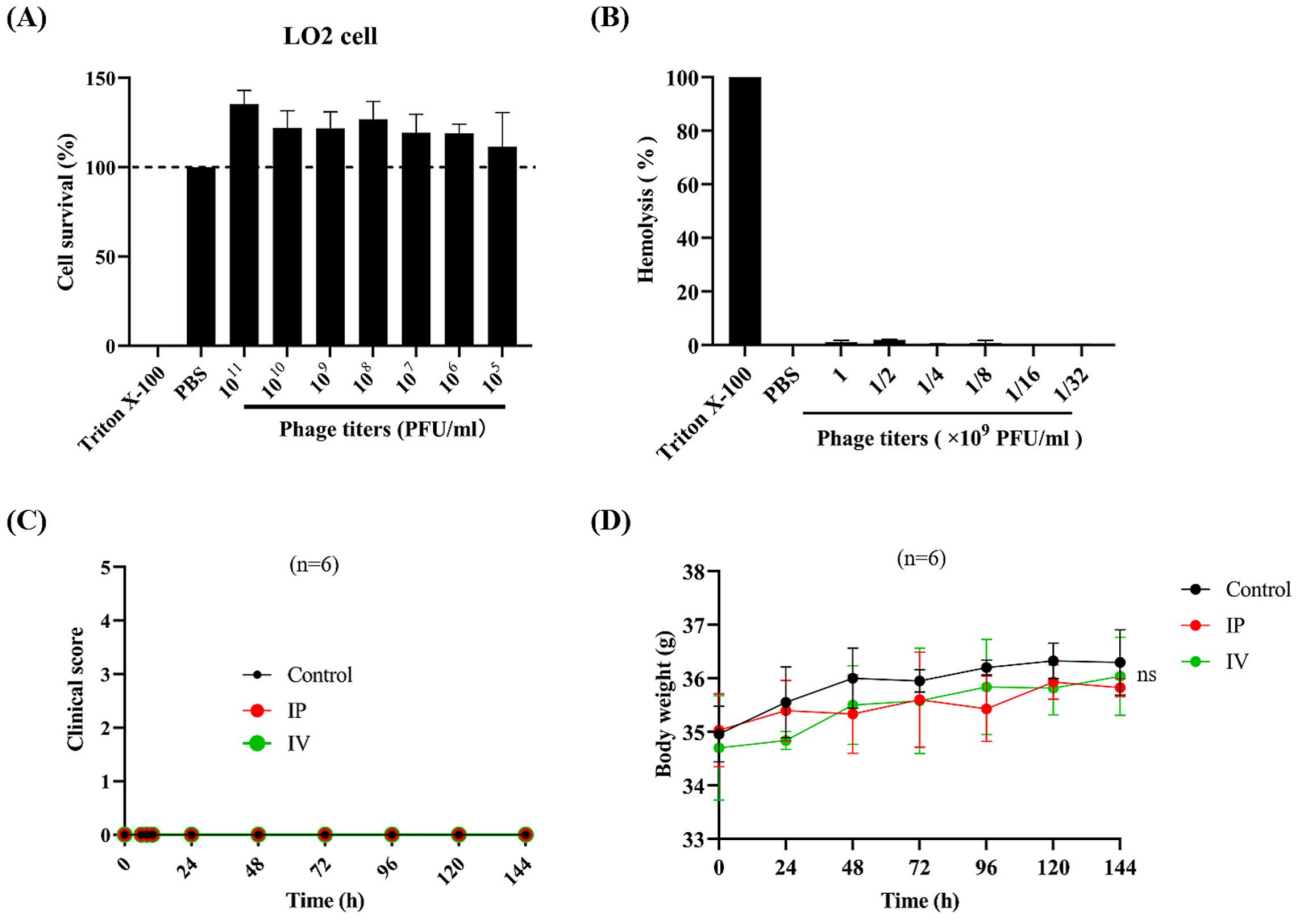
Safety evaluation of phage vB_EfaS-1017. **(A)** Cytotoxicity of vB_EfaS-1017. A CCK8 assay was conducted to determine the cytotoxicity of vB_EfaS-1017 by measuring the cell viability of L02 cells in the presence of vB_EfaS-1017. PBS and Triton X-100 (0.1%) were used as negative and positive controls, respectively. **(B)** Hemolytic activity of vB_EfaS-1017. Hemoglobin release from red blood cells (RBCs) was measured. PBS and Triton X-100 (0.1%) were used as negative and positive controls, respectively. **(C,D)** *In vivo* acute toxicity in the mouse model. Phage vB_EfaS-1017 was administered to mice via intraperitoneal (IP) or intravenous (IV) injection once daily for a week. PBS was used as a negative control. The clinical symptoms and body weight of the mice were monitored throughout the study. The statistical difference in body weights among the three groups was analyzed using the two-way ANOVA. ns, non-significant.

The cytotoxicity of phage vB_EfaS-1017 was evaluated using the CCK8 assay. This phage was purified by CsCl gradient centrifuge and dialysis. Even at a high concentration of 1 × 10^11^ PFU/mL, vB_EfaS-1017 did not exhibit any adverse effects on LO2 cells (Figure 6A). Additionally, a concentration of 1 × 10^9^ PFU/mL of vB_EfaS-1017 did not cause lysis of red blood cells (Figure 6B). These data suggest that vB_EfaS-1017 is safe for use *in vitro*.

To assess *in vivo* safety, phage vB_EfaS-1017 was administered to mice via either intraperitoneal or intravenous injection, once daily for a week. The clinical symptoms of the mice were monitored, and the health scores indicated that the mice remained in a healthy state with no observable symptoms (Figure 6C). Additionally, the body weights of mice injected with the phage solution showed no significant changes throughout the week (Figure 6D). At the endpoint of 144 h post phage injection, orbital blood was collected from each group. Cytokine analysis revealed no significant differences in the levels of IL-1β, IL-6, or TNF-*α* between the control and both the IP and IV treatment groups (Supplementary Figure S2). Additionally, blood analysis performed using an automated clinical chemistry analyzer revealed no significant differences in liver function of mice between the control and both the IP and IV treatment groups (Supplementary Table S3). Therefore, vB_EfaS-1017 is demonstrated to be safe *in vivo*.

### 3.6 Phage vB_EfaS_10–17 persists longer in the spleen of mice

**Figure 7:**
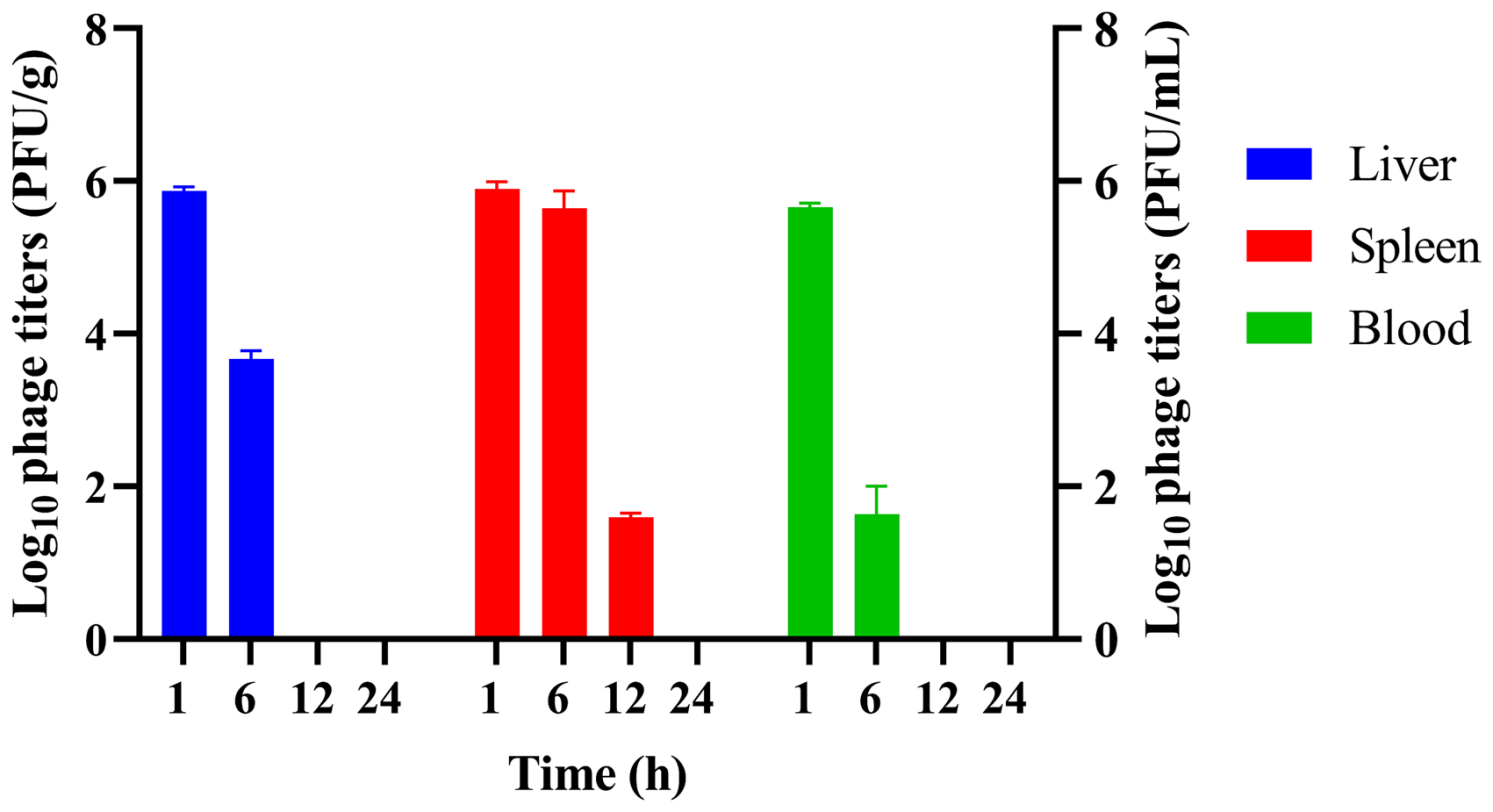
Determination of phage titer and distribution *in vivo.* Phage titers were measured in the liver, spleen, and blood of mice injected with vB_EfaS-1017 at various time points post-injection.

To investigate the distribution and concentration of phage vB_EfaS-1017 *in vivo*, KM mice were injected intraperitoneally with 2 × 10^8^ PFU per mouse of the phage. At 1 h post-injection, high concentrations of phages were detected in the liver, spleen, and blood. By 6 h post-injection, the phage titers had decreased rapidly in the blood and liver, although they remained elevated in the spleen. At 12 h post-injection, the phages were no longer detectable in the liver and blood, while a titer of 1 × 10^2^ PFU/g persisted in the spleen (Figure 7). These findings suggest that phages may not persist for extended periods in mice in the absence of bacterial hosts, yet they exhibit a longer persistence in the spleen. The detailed mechanism underlying this phenomenon remains unclear.

### 3.7 Combination of phage and levofloxacin is superior to phage alone against *E. faecalis* bacteremia in mouse

**Figure 8:**
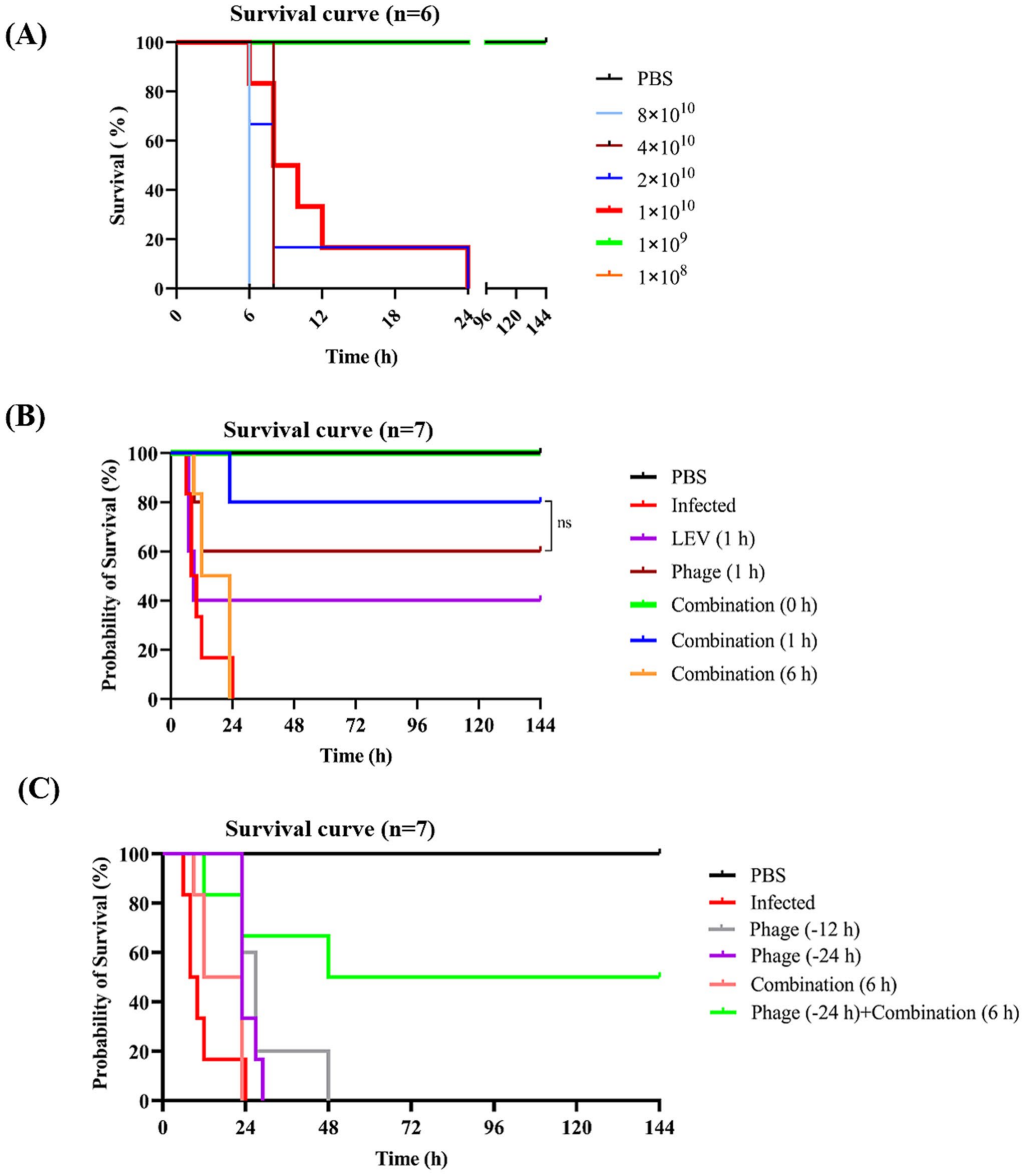
Survival curves of mice subjected to various treatments. **(A)** Establishment of a bacteremia mouse model. Mice were injected intraperitoneally with different doses of *E. faecalis* 10–17, with PBS serving as a negative control. **(B)** Efficacy evaluation of different treatment regimens. Infected mice were treated with phage vB_EfaS-1017, levofloxacin, or a combination of both. The optimal timing for combinatory treatment was also assessed. **(C)** Survival analysis of prophylactic administration of phage. Survival curves were analyzed using the Kaplan–Meier method in GraphPad Prism 9. ns, non-significant.

To establish a bacteremia model, KM mice were infected intraperitoneally with various doses of *E. faecalis* 10–17. All six mice injected with 1 × 10^10^ CFU per mouse died within 24 h. Infection with higher bacterial doses resulted in more rapid mortality. In contrast, mice infected with 1 × 10^9^ CFU or less per mouse exhibited a brief period of illness post-infection but gradually recovered and survived (Figure 8A). Consequently, 1 × 10^10^ CFU per mouse was selected to establish an acute infection model of *E. faecalis* 10–17.

At 1 h post-infection (POI), mice were treated with phage vB_EfaS-1017 alone, levofloxacin (LEV), or a combination of both. A 6-h POI checkpoint was used to euthanize the mice and assess bacterial load in the liver, spleen, and blood. Compared to the untreated infected group, LEV alone did not significantly reduce bacterial levels in any of the organs. In contrast, the combination of phage and LEV exhibited the most effective bactericidal activity, significantly reducing bacterial loads (Supplementary Figure S3). Consistently, at 144 h POI, the survival rates for the phage, LEV, and combination treatment groups were 60, 40, and 80%, respectively (Figure 8B), demonstrating that the combination therapy is more effective than either phage or LEV alone.

Furthermore, to determine the optimal timing for combination therapy, treatments were administered immediately after infection (0 h) and at 6 h POI. We found that immediate administration of the combination treatment protected 100% of the infected mice. However, when treatment was delayed until 6 h POI, all mice died of the infection (Figure 8B). These results suggest that early intervention is crucial for the effectiveness of phage therapy in combating bacteremia.

To investigate the cause of death in mice treated with the combination therapy, we dissected a dead mouse and harvested its liver. Bacteria isolated from the liver were cultured, and 30 colonies were picked and identified using MALDI-TOF mass spectrometry. All 30 colonies were identified as *E. faecalis*, and 10 of these exhibited resistance to phage vB_EfaS-1017 (Supplementary Figure S4). The levofloxacin susceptibility of all tested colonies remained unchanged (data not shown). These data suggest that the mortality in mice is likely due to the emergence of phage-resistant *E. faecalis*.

### 3.8 Prophylactic administration of phage offers a time window for delayed treatment

To investigate the protective effect of prophylactic phage administration, we injected mice with phage at either 12 h or 24 h before bacterial challenge. However, all mice that received prophylactic phage administration alone succumbed to the infection. However, 50% of the mice that received prophylactic phage administration followed by delayed combination treatment survived (Figure 8C). These data suggest that prophylactic administration of phage alone does not provide protection. However, prophylactic administration of phage may offer a critical time window for delayed treatment.

**Figure 9:**
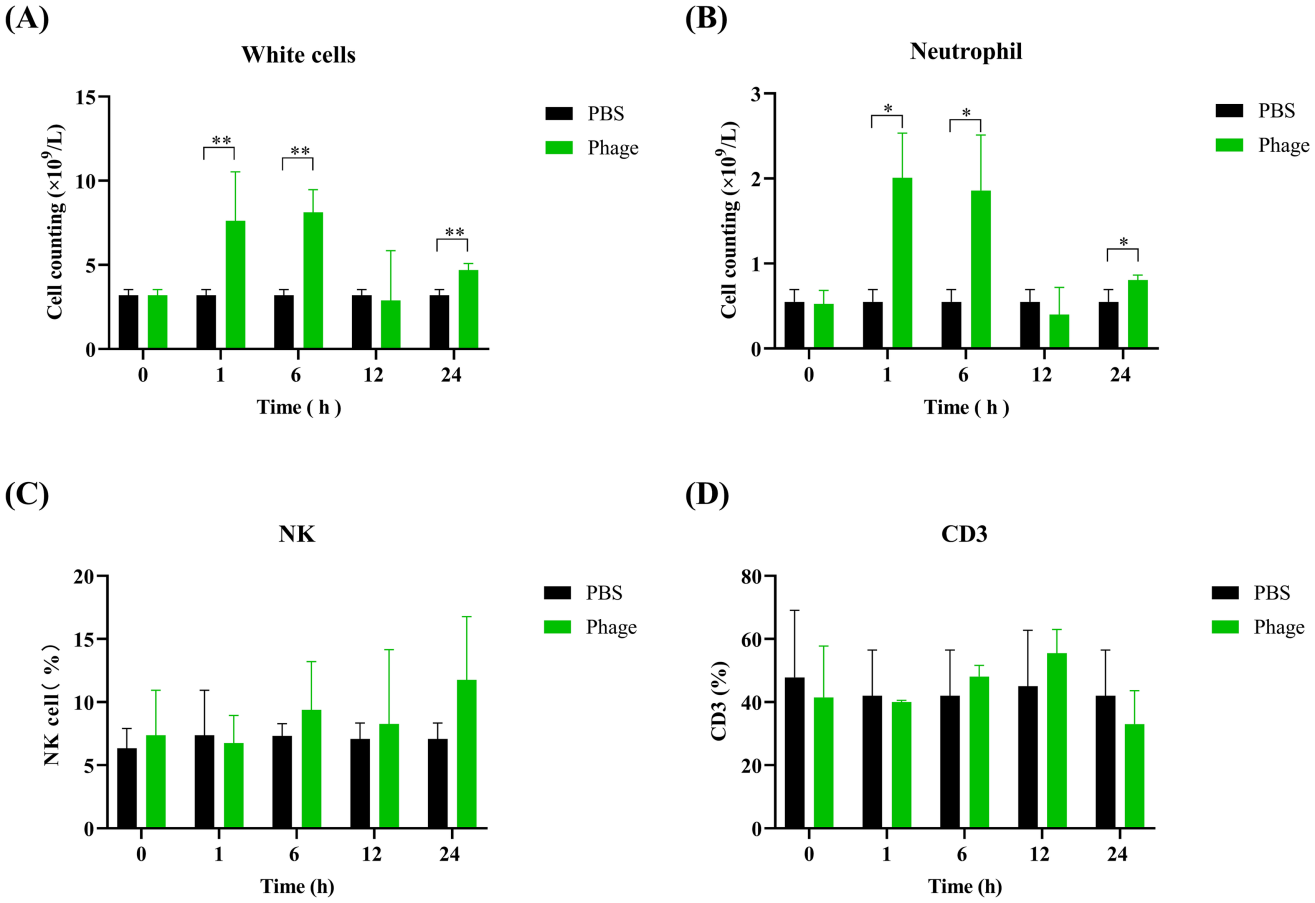
Analysis of immune cell populations in mice prophylactically administered with phage vB_EfaS-1017. White blood cell **(A)** and neutrophil **(B)** counts were determined via complete blood count (CBC) examination using the Sysmex XN-2800 hematology analyzer. NK cells **(C)** and CD3+ T cells **(D)** were quantified using flow cytometry after labeling with FITC-anti-CD3 and APC-anti-HLA-DR antibodies. Data were analyzed using the one-way ANOVA with multiple comparisons. Statistical significance is indicated as follows: **p < 0.001; *p < 0.05.

To elucidate the protective mechanism of prophylactic phage administration, mice were injected with phage, and blood samples were collected at various time points. Blood cell counts revealed that white blood cell and neutrophil counts were elevated in the phage-treated group compared to the control group at 1 h and 6 h post-injection. However, no significant differences were observed at 24 h post-injection (Figures 9A,B). Additionally, flow cytometry analysis showed no significant differences in NK cell and CD3 cell counts between the phage-treated and control groups (Figures 9C,D). These findings suggest that while prophylactic phage administration influences initial immune cell responses, the precise mechanism of protection remains unclear.

### 3.9 Phage-LEV combination treatment influences mouse gut microbiota

**Figure 10:**
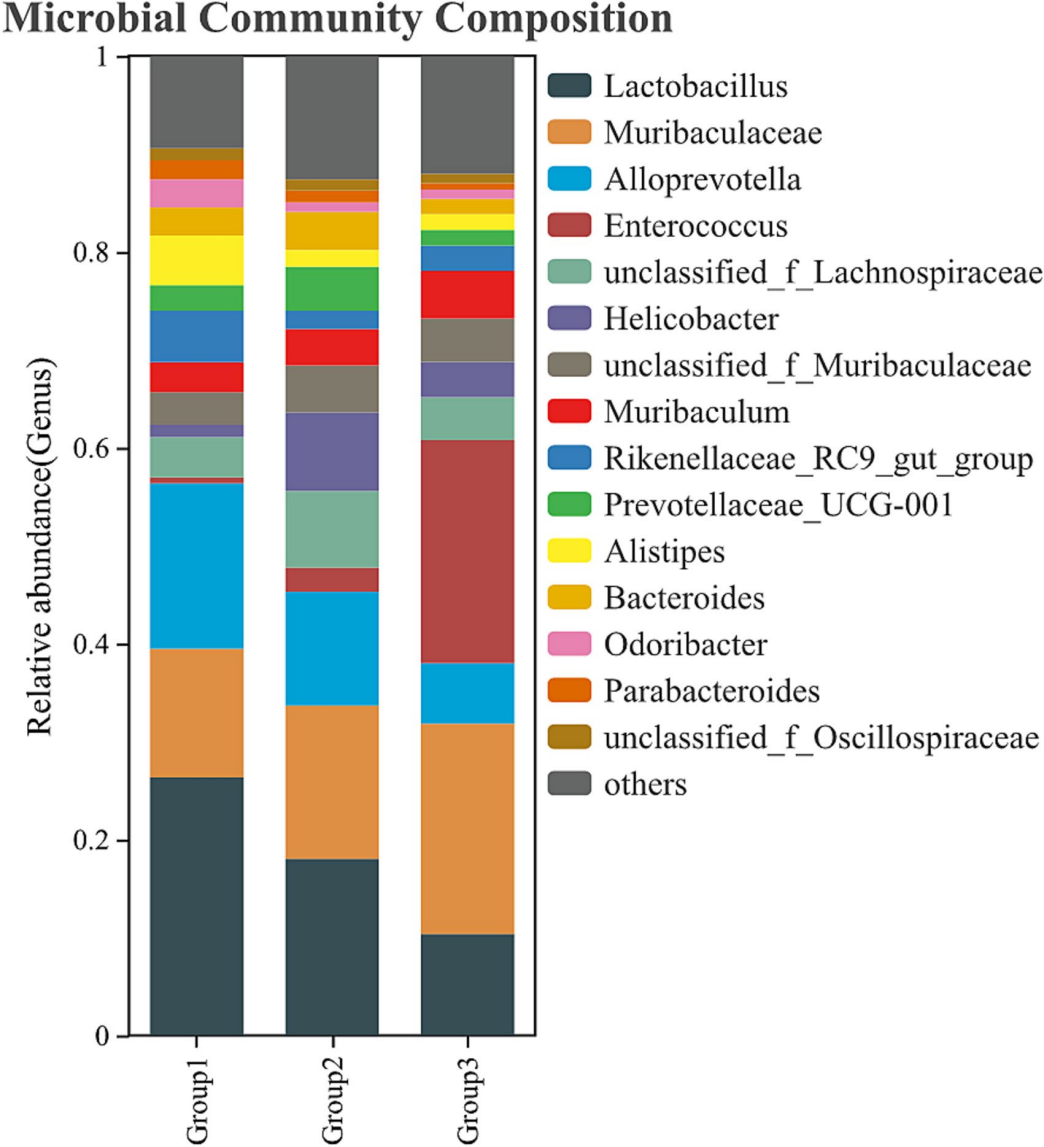
Composition of the gut microbiota. Fecal samples were collected from the colons of mice, and bacterial DNA was isolated for 16S rDNA gene sequencing. The relative abundance of intestinal bacteria at the genus level is presented. Group 1: uninfected control; Group 2: combination treatment group; Group 3: infected but untreated group. Each group consisted of 6 mice.

Infection with *E. faecalis* disrupted the normal balance of the gut microbiota, with *Enterococcus* genus becoming the most abundant bacterial group in the infected group. Additionally, bacteria in the *Muribaculaceae* genus also increased significantly. In contrast, the abundance of bacteria from the genera *Lactobacillus*, *Alloprevotella*, and *Helicobacter* decreased markedly. However, administration of the phage-LEV combination therapy resulted in a marked restoration of the microbiota composition. The abundance of *Enterococcus* and *Muribaculaceae* decreased, approaching the normal levels observed in the uninfected control mice. Furthermore, the abundance of *Lactobacillus* and *Alloprevotella* significantly increased (Figure 10 and Supplementary Table S4). These results suggest that *E. faecalis* infection induces significant shifts in the gut microbiota, and that the combination treatment with phage and LEV is capable of reversing these disruptions, restoring the microbiota to a more balanced state.

## 4 Discussion

In this study, we characterized a novel *Enterococcus* phage vB_EfaS-1017 and evaluated its potential as a therapeutic agent against *E. faecalis* infections. Our results demonstrated that vB_EfaS-1017 possesses a robust bactericidal activity and a significant ability to inhibit and eradicate biofilms, which are often resistant to conventional antibiotics. Whole genome sequencing revealed that vB_EfaS-1017 has a circular double-stranded DNA genome of 40,766 bp, with a GC content of 34.82% and 65 predicted open reading frames (ORFs). The BLASTp analysis showed that the highest similar phage was the phage vB_EfaS_SRH2, only with 91.19% identity and 79% coverage. The absence of virulence and antibiotic resistance genes in the phage genome, as confirmed using the VFDB and CARD analyses, underscores its potential safety for therapeutic applications. *In vitro* assays demonstrated that vB_EfaS-1017 could rapidly kill *E. faecalis* cells, achieving a 95% reduction within the first hour of exposure. However, a small fraction of bacteria persisted, likely due to the emergence of phage-resistant mutants. The phage also exhibited significant biofilm inhibition and eradication capabilities, which are crucial for treating chronic and biofilm-associated infections. Notably, phage activity against biofilms was dose-independent, highlighting its potential efficacy even at lower concentrations.

In our bacteremia mouse model, all mice infected with *E. faecalis* died within 24 h without any treatment. *E. faecalis* is known to produce various toxic secreted products, such as cytolysin, which can cause tissue damage and increase mortality (Jett et al., 1994). Treatment with phage alone rescued 60% of the mice, while treatment with levofloxacin alone rescued 40%. However, the combination of both treatments rescued 80% of the mice, indicating that a combinatory regimen of phage and antibiotic is more effective. This finding is consistent with previous studies. Lev et al. reported that the *E. faecium* bacteriophage 113 exhibited synergy with daptomycin and ampicillin in eliminating MDR *E. faecium* biofilm *in vitro* (Lev et al., 2022). Similarly, a combination of *E. faecalis* bacteriophage vB_EfaM_LG1 and cefotaxime showed a more significant effect in disrupting biofilms than a single treatment (Song et al., 2021). Furthermore, combined bacteriophage-antibiotic treatment has been shown to significantly reduce bacterial load and inflammatory levels in mice infected with MDR *E. faecalis* (Gelman et al., 2018). An additional advantage of phage-antibiotic combinations is their potential to prevent the development of phage resistance (Chanishvili, 2016; Łusiak-Szelachowska et al., 2022). In a previous study, we found no phage-resistant strains isolated from the liver of infected and dead mice treated with a combination of phage ФAb4B and ciprofloxacin (Wang et al., 2024). However, in this study, approximately 30% of colonies isolated from the liver of infected and dead mice treated with the phage-antibiotic combination were phage-resistant. These findings suggest that the efficacy of preventing phage resistance may vary depending on the bacterial species. Further investigation *in vivo* is warranted to explore the efficacy of phage-antibiotic combination regimens in preventing phage resistance. The observed synergy between phage vB_EfaS-1017 and levofloxacin underscores the potential of integrated antimicrobial strategies to enhance treatment outcomes against *E. faecalis* infections. Future research should focus on optimizing dosing regimens, elucidating the mechanisms underlying phage-antibiotic interactions, and conducting clinical trials to validate these findings in human subjects. In conclusion, our study demonstrates that phage vB_EfaS-1017, especially when used in combination with antibiotics, holds significant promise as a therapeutic agent against *E. faecalis* infections.

It is well-established that phages can be used prophylactically to prevent infections. For example, the prophylactic use of a single bacteriophage, Phi_1, successfully controlled cholera in an infant rabbit model (Bhandare et al., 2019). Similarly, *in vivo* oral administration of isolated phages reduced mortality in day-old chicks infected with avian pathogenic *Escherichia coli* (Jhandai et al., 2024). Another study demonstrated that prophylactic delivery of a bacteriophage cocktail in feed significantly reduced *Salmonella* colonization in pigs (Thanki et al., 2022). These studies across various animal models indicate that phages can effectively control bacterial infections when used prophylactically. In our study, prophylactic administration of phage 24 h before infection did not rescue the infected mice. However, the combination of prophylactic phage administration and delayed treatment with a combinatory regimen rescued 60% of the mice, compared to the 100% mortality observed with delayed treatment alone. This finding is consistent with our previous results in an *Acinetobacter baumannii*-infected mouse bacteremia model, where the combination of prophylactic phage use and delayed treatment saved 100% of the infected mice (Wang et al., 2024). Thus, the current study demonstrates that prophylactic phage administration can provide a valuable time window for delayed treatment.

An important consideration is the timing of phage administration in prophylaxis, as phages do not persist for long *in vivo* without a bacterial host. Previous studies have shown that virulent *S. aureus* A5/L bacteriophages administered intraperitoneally 30 min before infection increased the percentage of circulating neutrophils and immature cells from the myelocytic and lymphocytic lineages in *S. aureus*-infected mice (Zimecki et al., 2009). In our study, phages were completely cleared from the mice after 12 h of injection. Consistently, phages administered 24 h before infection did not provide any protective effect. This raises the question of what happens after 24 h of phage administration and how this early prophylaxis, combined with delayed treatment, results in different outcomes compared to delayed treatment alone. We analyzed humoral cells 24 h after phage administration and found no differences in NK cell and neutrophil counts compared to the control group. The counts of CD3+ cells were also similar. Therefore, the precise mechanism of how early prophylactic use of phage provides a protective effect remains unclear and warrants further investigation. In clinical practice, the narrow spectrum of phages necessitates sufficient time for identifying the causative pathogen and determining phage sensitivity. Consequently, timely phage therapy can be challenging. It is crucial to investigate how prophylactic phage administration affects the immune response and whether its efficacy is species-specific.

The gut microbiota is a complex and dynamic ecosystem, which plays a critical role in maintaining host health by aiding in digestion, immune regulation, and pathogen defense. Disruptions to the microbiota, caused by infections or antimicrobial treatments, can result in dysbiosis, which may contribute to various diseases. In this study, we investigated the impact of phage therapy, in combination with levofloxacin, on the gut microbiota of mice infected with *E. faecalis*. Our findings indicate that *E. faecalis* infection alone caused significant alterations to the gut microbiota, consistent with previous studies showing that bacterial infections can disrupt microbial diversity (Cheng et al., 2017). Specifically, *Enterococcus* became the dominant genus in the microbiota of infected mice, a finding that aligns with the pathogenic role of *E. faecalis* in dysbiosis during infection. Additionally, the decrease in Lactobacillus, Alloprevotella, and Helicobacter, all of which are beneficial gut microbes involved in maintaining gut health, further supports the notion that infection-induced dysbiosis impairs the microbiome’s protective functions (DeGruttola et al., 2016). Phage therapy, particularly the combination of phage vB_EfaS-1017 and LEV, demonstrated a remarkable ability to reverse these infection-induced changes. Administration of this combination therapy restored the abundance of beneficial bacteria, such as *Lactobacillus* and *Alloprevotella*, while reducing the dominance of *Enterococcus* and *Muribaculaceae*. These results are consistent with previous reports, suggesting that phage therapy may not only target the pathogenic bacteria but also help maintain or restore the ecological balance of the microbiota by selectively reducing the abundance of the infecting pathogen without causing broad-spectrum disruption to other microbial communities (Chen et al., 2023).

## 5 Conclusion

Overall, we isolated and characterized a novel lytic phage of vancomycin-resistant *E. faecalis*, vB_EfaS-1017. This phage possesses a circular double-stranded DNA genome of 40,766 bp and shares 91.19% identity and 79% coverage with *Enterococcus* phage vB_EfaS_SRH2. Despite its narrow host spectrum, vB_EfaS-1017 exhibited robust bactericidal activity *in vitro* and demonstrated a significant capacity to inhibit and eradicate biofilms. Additionally, vB_EfaS-1017 showed synergy with levofloxacin in inhibiting bacterial growth. Safety evaluations confirmed that the phage is safe both *in vitro* and *in vivo*, with a persistence time of less than 12 h in the spleen of mice.

In a mouse bacteremia model, the administration of phage alone rescued 60% of mice infected with vancomycin-resistant *E. faecalis*, while a combination of phage and levofloxacin saved 80%. Prophylactic administration of phage 24 h before infection did not protect any mice. However, a combination of prophylactic phage administration and delayed treatment rescued 60% of mice, compared to 100% mortality in the group receiving delayed treatment alone. Phage therapy can not only target the pathogenic bacteria but also help maintain or restore the ecological balance of the microbiota. Our study underscores the potential of phage-antibiotic combinations as a more effective therapeutic approach against bacterial infections. Prophylactic use of phage can provide a valuable time window for delayed treatment. Further research and preclinical trials are needed to optimize phage therapy protocols and better emulate the conditions under which phage therapy is most likely to be beneficial for human use.

## Data Availability

The datasets presented in this study can be found in online repositories. The names of the genomic DNA sequence data of phage vB_EfaS-1017 presented are deposited in the GenBank with accession numbers PP894992.

## Supplementary material

SUPPLEMENTARY FIGURE S1 Evolutionary tree of phage vB_EfaS-1017 based on its terminase large subunit protein sequence (A) and major capsid protein sequence (B). The evolutionary relationships were conducted in MEGA7 using the Neighbor-Joining method. The bootstrap consensus tree inferred from 500 replicates is taken to represent the evolutionary history of the taxa analyzed.

SUPPLEMENTARY FIGURE S2 Evaluation of cytokine levels *in vivo*. Orbital blood was collected from mice at 144 h post-phage injection to assess the levels of IL-1β, IL-6, and TNF-α by the ELISA assay.

SUPPLEMENTARY FIGURE S3 Bacterial loads in the liver, spleen, and blood of mice at the 6-hour POI checkpoint. The different treatment regimens were initiated 1 hour POI. Data were analyzed using one-way ANOVA with multiple comparisons. Statistical significance is indicated as follows: ***p* < 0.001; **p* < 0.05.

SUPPLEMENTARY FIGURE S4 Heatmap of phage susceptibility of bacteria isolated from dead mouse treated by combinatory regimen. Dark blue, large and clear plaque; light blue, vague plaque; white, no plaque.
