# Supplementary figures and images for: Prophylactic phage administration provides a time window for delayed treatment of vancomycin-resistant Enterococcus faecalis in a murine bacteremia model
Source: Front Microbiol. 2025 Jan 24;15:1504696. doi: 10.3389/fmicb.2024.1504696 (PMC11802572; doi:10.3389/fmicb.2024.1504696)

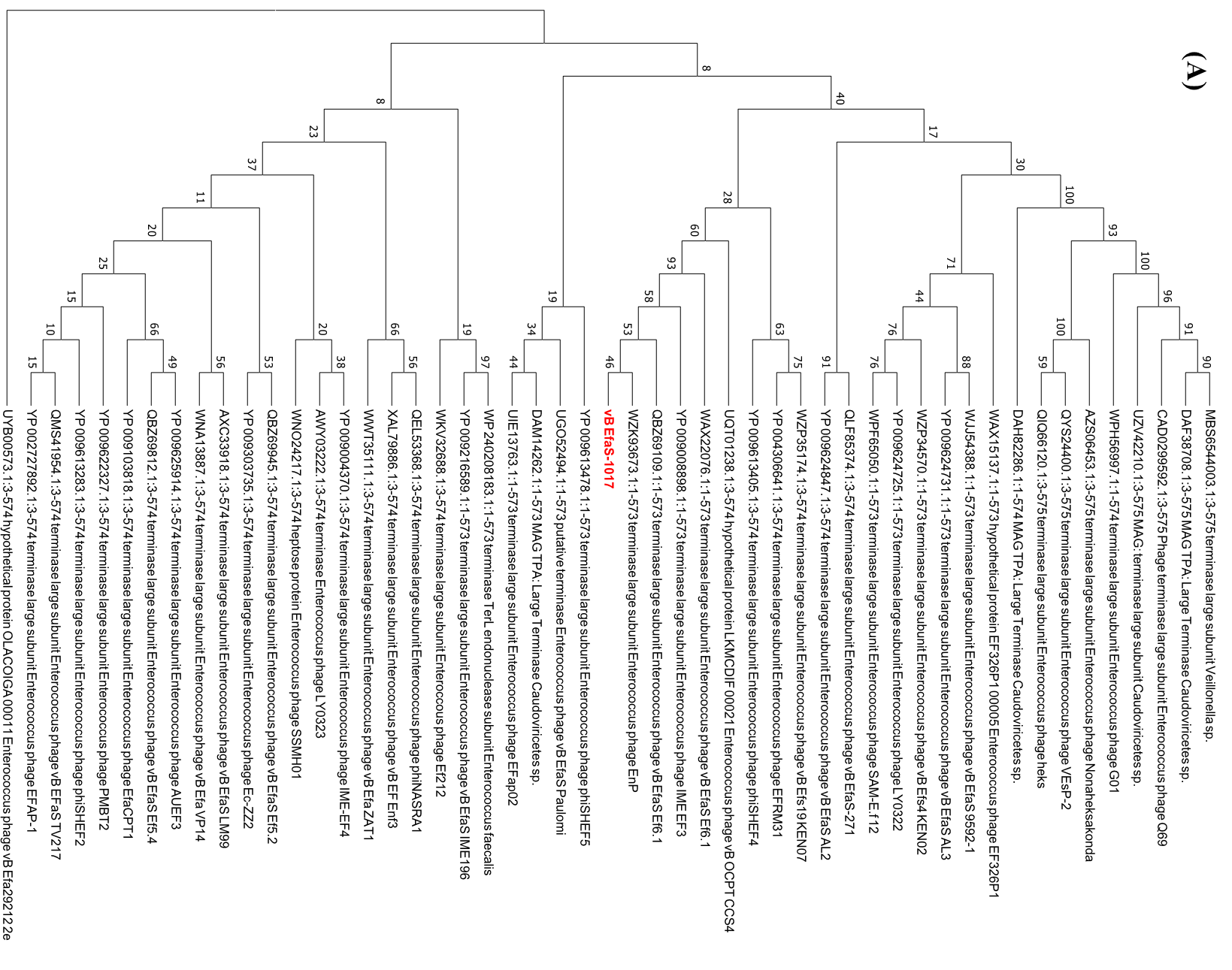

Supplement: SUPPLEMENTARY FIGURE S1 — Evolutionary tree of phage vB_EfaS-1017 based on its terminase large subunit protein sequence (A) and major capsid protein sequence (B). The evolutionary relationships were conducted in MEGA7 using the Neighbor-Joining method. The bootstrap consensus tree inferred from 500 replicates is taken to represent the evolutionary history of the taxa analyzed. [file Image_1.TIF]

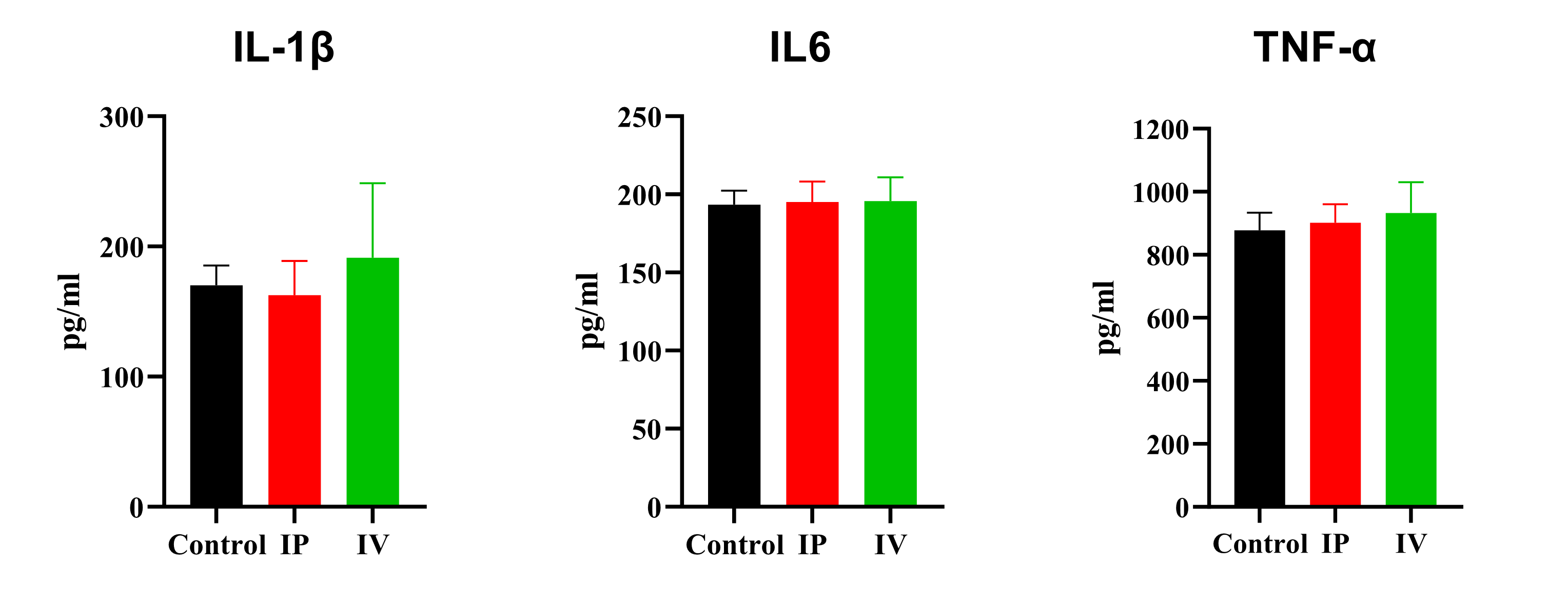

Supplement: SUPPLEMENTARY FIGURE S2 — Evaluation of cytokine levels in vivo. Orbital blood was collected from mice at 144 h post-phage injection to assess the levels of IL-1β, IL-6, and TNF-α by the ELISA assay. [file Image_2.PNG]

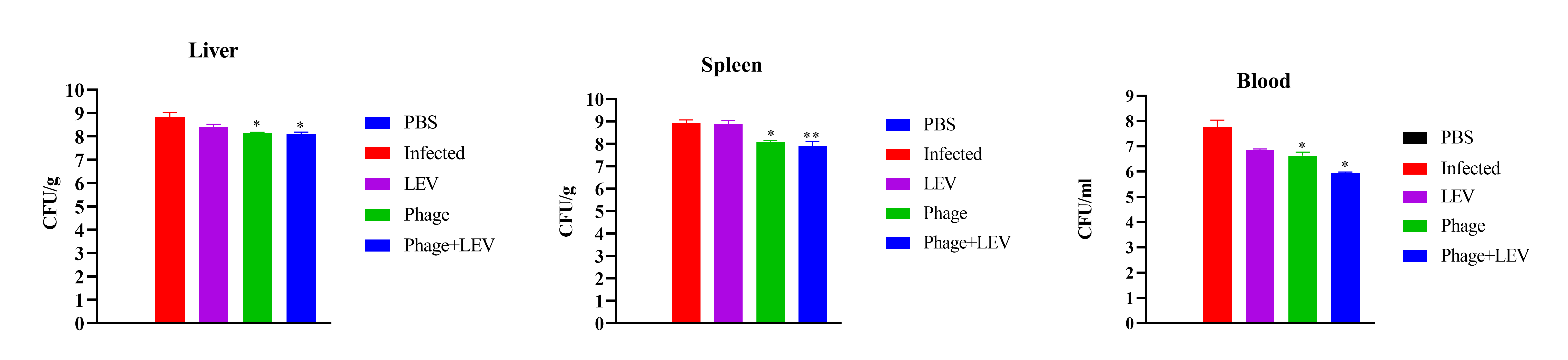

Supplement: SUPPLEMENTARY FIGURE S3 — Bacterial loads in the liver, spleen, and blood of mice at the 6-hour POI checkpoint. The different treatment regimens were initiated 1 hour POI. Data were analyzed using one-way ANOVA with multiple comparisons. Statistical significance is indicated as follows: **p < 0.001; *p < 0.05. [file Image_3.TIF]

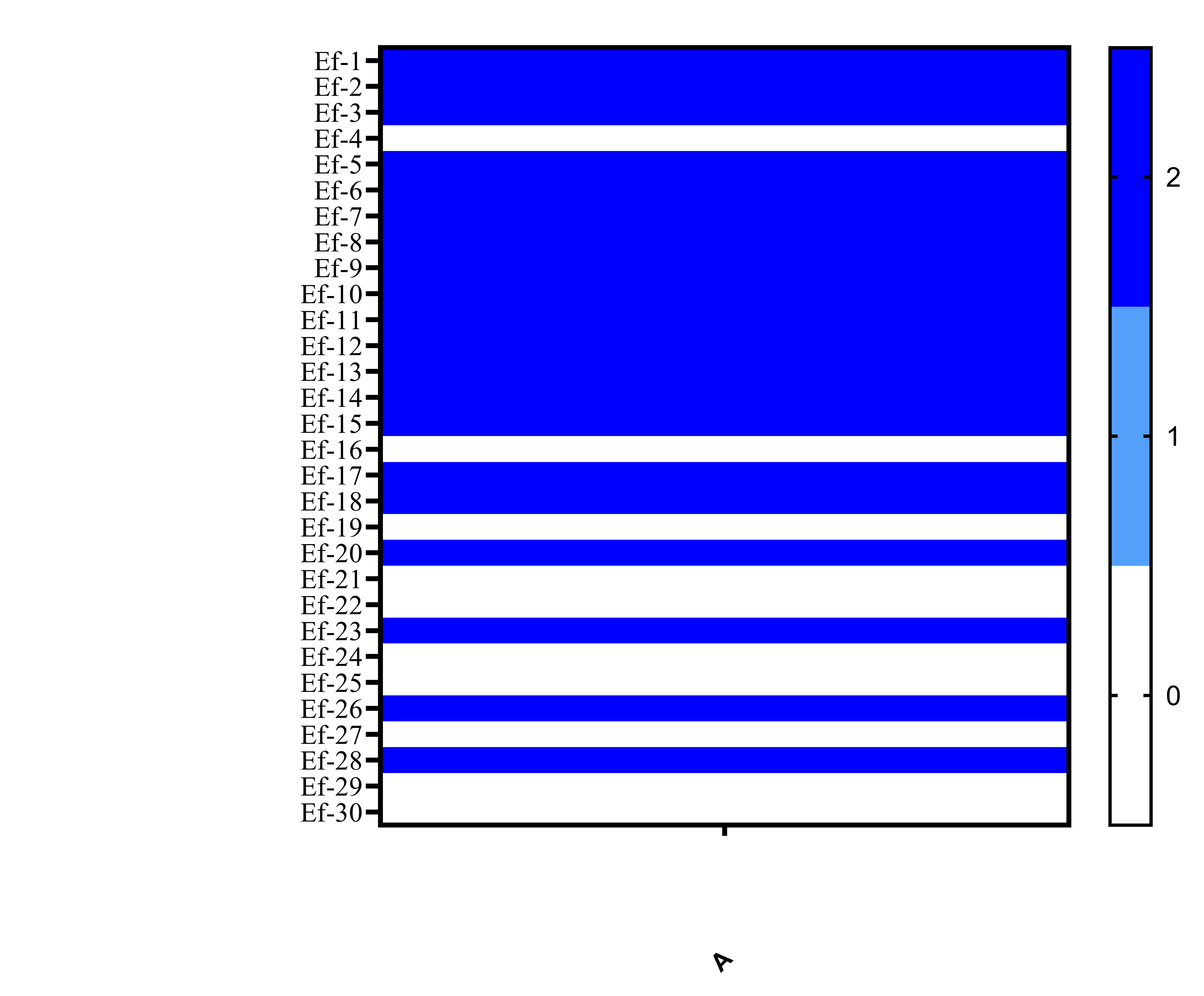

Supplement: SUPPLEMENTARY FIGURE S4 — Heatmap of phage susceptibility of bacteria isolated from dead mouse treated by combinatory regimen. Dark blue, large and clear plaque; light blue, vague plaque; white, no plaque. [file Image_4.TIF]
