# Supplementary material for: Prophylactic phage administration provides a time window for delayed treatment of vancomycin-resistant Enterococcus faecalis in a murine bacteremia model
Source: Front Microbiol. 2025 Jan 24;15:1504696. doi: 10.3389/fmicb.2024.1504696 (PMC11802572; doi:10.3389/fmicb.2024.1504696)
Supplement: Supplementary file 5 [file Table_1.DOCX]

**Table S1. Bacteria used in this study and their susceptibility to phage vB_EfaS-1017.**

| **Bacteria** | **Characteristics** | **Phage sensitivity** | **Sources** |
| --- | --- | --- | --- |
| *Enterococcus faecalis* 7-11 | vancomycin-resistant | - | the Second Hospital of Nanjing |
| *Enterococcus faecalis* 7-12 | vancomycin-resistant | - | the Second Hospital of Nanjing |
| *Enterococcus faecalis* 10-7 | vancomycin-resistant | - | the Second Hospital of Nanjing |
| *Enterococcus faecalis* 10-17 | vancomycin-resistant | **++** | the Second Hospital of Nanjing |
| *Enterococcus faecalis* 10-28 | vancomycin-resistant | **++** | the Second Hospital of Nanjing |
| *Enterococcus faecalis* 10-37 | vancomycin-resistant | - | the Second Hospital of Nanjing |
| *Enterococcus faecalis* 10-58 | vancomycin-resistant | **++** | the Second Hospital of Nanjing |
| *Enterococcus faecalis* 7-18 | vancomycin-resistant | - | the Second Hospital of Nanjing |
| *Enterococcus faecalis* 7-19 | vancomycin-resistant | - | the Second Hospital of Nanjing |
| *Enterococcus faecalis* 7-20 | vancomycin-resistant | - | the Second Hospital of Nanjing |
| *Enterococcus faecalis* 7-21 | vancomycin-resistant | - | the Second Hospital of Nanjing |
| *Enterococcus faecium* 10-3 | vancomycin-resistant | - | the Second Hospital of Nanjing |
| *Enterococcus faecium* 11-31 | vancomycin-resistant | - | the Second Hospital of Nanjing |
| *Enterococcus faecium* 11-34 | vancomycin-sensitive | - | the Second Hospital of Nanjing |
| *Enterococcus faecium* 11-38 | vancomycin-resistant | - | the Second Hospital of Nanjing |
| *Enterococcus faecium* 11-39 | vancomycin-resistant | - | the Second Hospital of Nanjing |
| *Enterococcus faecium* 11-68 | vancomycin-resistant | - | the Second Hospital of Nanjing |
| *Streptococcus pneumoniae* 12-5 | vancomycin-sensitive | - | the Second Hospital of Nanjing |
| *Streptococcus agalactiae* 12-6 | vancomycin-sensitive | - | the Second Hospital of Nanjing |
| *Staphylococcus aerues* 11-46 | methicillin-resistant | - | the Second Hospital of Nanjing |
| *Staphylococcus aerues* 11-47 | methicillin-resistant | - | the Second Hospital of Nanjing |
| *Esherichia coli* 13-1 | carbapenem-resistant | - | the Second Hospital of Nanjing |
| *Esherichia coli* 13-2 | carbapenem-sensitive | - | the Second Hospital of Nanjing |
| *Acinetobacter baumannii* 10-1 | carbapenem-resistant | - | the Second Hospital of Nanjing |
| *Acinetobacter baumannii* 10-2 | carbapenem-sensitive | - | the Second Hospital of Nanjing |
| *Pseudomonas aeruginosa* 1-1 | carbapenem-resistant | - | the Second Hospital of Nanjing |
| *Pseudomonas aeruginosa* 1-2 | carbapenem-resistant | - | the Second Hospital of Nanjing |
