# Supplementary material for: Prophylactic phage administration provides a time window for delayed treatment of vancomycin-resistant Enterococcus faecalis in a murine bacteremia model
Source: Front Microbiol. 2025 Jan 24;15:1504696. doi: 10.3389/fmicb.2024.1504696 (PMC11802572; doi:10.3389/fmicb.2024.1504696)
Supplement: Supplementary file 6 [file Table_2.DOCX]

**Table S1. Minimal inhibitory concentrations (MIC) of different antimicrobials (μg/ml)**

| **Antimicrobial agents** | ***E. faecalis* 10-17** |
| --- | --- |
| Vancomycin | 4 |
| Tetracycline | 256 |
| Chloramphenicol | 128 |
| Carbenicillin | 128 |
| Ciprofloxacin | 128 |
| Levofloxacin | 256 |
| Erythromycin | 256 |
| Linezolid | 1 |
| Cefoxitin | 256 |
| Cephalothin/sulbactam | 512 |
| COG1410  Ampicillin | 256  258 |
